# Nanozyme‐Reinforced miR‐197‐3p Delivery Resets Metabolic and Senescence Pathways to Rejuvenate Osteoarthritic Cartilage

**DOI:** 10.1002/advs.76364

**Published:** 2026-07-04

**Authors:** Xuejie Cai, Zehui Lv, Chen Zhang, Xingdong Yang, Ruoying Wang, Yixin Bian, Jiawei Xu, Han Wang, Yingjie Wang, Long Bai, Jiacan Su, Xisheng Weng

**Affiliations:** ^1^ Peking Union Medical College Hospital Chinese Academy of Medical Science and Peking Union Medical College Beijing China; ^2^ Department of Orthopedic Surgery State Key Laboratory of Complex Severe and Rare Diseases Beijing China; ^3^ MedEng‐X Institutes Shanghai University Shanghai China; ^4^ Organoid Research Center Institute of Translational Medicine Shanghai University Shanghai China; ^5^ National Center for Translational Medicine (Shanghai) SHU Branch Shanghai University Shanghai China; ^6^ Wenzhou Institute of Shanghai University Wenzhou China; ^7^ Department of Orthopedics Xinhua Hospital Affiliated to Shanghai Jiao Tong University School of Medicine Shanghai China

**Keywords:** hydrogel microspheres, miR‐197‐3p delivery, mitochondrial reprogramming, osteoarthritis, redox microenvironment modulation

## Abstract

Osteoarthritis (OA) is a progressive and disabling joint disease driven by oxidative stress, chondrocyte senescence and extracellular matrix (ECM) degradation, yet lacks effective disease‐modifying treatments. In this study, we identified miR‐197‐3p as a previously unrecognized, cartilage‐protective miRNA significantly downregulated in both aged and osteoarthritic cartilage. Functional studies revealed that miR‐197‐3p restores ECM anabolism, suppresses senescence and directly targets G3BP1, a stress granule protein linked to redox imbalance and inflammatory signaling. To enable effective intra‐articular delivery, we engineered a multifunctional microsphere platform (miR/PBNP@Gel) by co‐encapsulating miR‐197‐3p and ultrasmall Prussian blue nanozymes (PBNPs) into GelMA hydrogel microspheres. This composite design synergistically enhances miRNA stability, facilitates cellular internalization and provides continuous reactive oxygen species (ROS) scavenging to protect mitochondrial function. miR/PBNP@Gel reversed mitochondrial dysfunction and senescence in OA chondrocytes, while promoting cartilage repair and joint function in vivo. Metabolomic profiling further revealed reprogramming of TCA cycle and antioxidant pathways. This work established miR‐197‐3p as a novel therapeutic regulator in OA and introduced a bioinstructive, injectable, and cell‐free strategy that integrates miRNA therapy and redox modulation for disease modification and cartilage regeneration.

## Graphical abstract

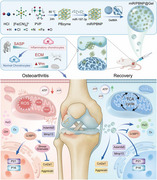

## 1 Introduction

Osteoarthritis (OA) is a prevalent and disabling degenerative joint disease marked by progressive articular cartilage deterioration, subchondral bone remodeling, and synovial inflammation [1]. Central to OA progression is the dysfunction of chondrocytes, the sole cellular component of cartilage, where an imbalance between anabolic and catabolic processes, driven by sustained oxidative stress, leads to the degradation of the extracellularmatrix (ECM) [2, 3, 4, 5, 6]. Accumulation of reactive oxygen species (ROS) impairs mitochondrial function and induces chondrocyte senescence, further aggravating joint inflammation through the secretion of senescence‐associated secretory phenotype (SASP) factors [7, 8]. As a result, OA affects over 500 million individuals globally, with increasing prevalence in aging populations [9, 10, 11]. However, existing clinical strategies remain palliative and can not reverse cartilage degeneration or correct the underlying redox and metabolic imbalances, thus driving the need for disease‐modifying and regeneration‐oriented therapeutics [12, 13].

Recent advances in RNA therapeutics have positioned microRNAs (miRNAs) as promising candidates for modulating OA‐associated gene networks [14, 15, 16, 17, 18, 19]. Multiple studies demonstrate miRNA delivery can attenuate cartilage degeneration and synovial inflammatory signaling in preclinical OA models. For example, circulating miR‐126‐3p has been identified as an OA‐associated biomarker, and its intra‐articular or systemic delivery significantly reduced disease severity by suppressing angiogenesis‐related pathways and secondary joint degeneration processes [20]. In addition, mesenchymal stem cell‐derived extracellular vesicles delivering miRNAs such as let‐7a‐5p have been shown to inhibit osteoclast activity and preserve subchondral bone and cartilage integrity in OA models [21]. Furthermore, miR‐26b‐5p delivered via macrophage‐derived exosomes reprograms macrophage polarization and suppresses chondrocyte hypertrophy, thereby alleviating synovitis and cartilage degeneration [22].

In this study, we conducted an integrated transcriptomic analysis using public databases and identified miR‐197‐3p as a previously unrecognized but mechanistically relevant miRNA, chosen for its consistently downregulated in both aged and osteoarthritic cartilage. Functional assays confirmed that miR‐197‐3p restoration enhances ECM synthesis, represses matrix‐degrading enzymes, and attenuates senescence phenotypes in chondrocytes. Notably, we uncovered G3BP1 as a novel direct target of miR‐197‐3p, linking it to redox stress response and cellular aging via stress granule signaling [23, 24]. These findings establish miR‐197‐3p not only as a diagnostic marker of chondrocyte aging but also as a potent molecular switch capable of reshaping the OA microenvironment. However, like many miRNA‐based therapies, its clinical application remains limited by rapid enzymatic degradation, low intra‐articular retention, and inefficient cellular uptake [25, 26]. To address these translational challenges, biomaterial‐assisted delivery platforms have emerged as a powerful tool to stabilize miRNA cargoes, enhance joint residency, and enable spatiotemporally controlled release.

Hydrogel carriers, such as gelatin methacryloyl (GelMA) microspheres and other injectable systems, have been widely explored for intra‐articular delivery in OA, improving drug retention and therapeutic efficacy in vivo [27, 28, 29, 30, 31, 32]. Recent advances further highlight their functional versatility. Injectable piezoelectric hydrogels can generate localized bioelectrical cues under external stimulation, thereby promoting stem cell recruitment and osteochondral regeneration [33]. Notably, biofunctional hydrogels enabling sustained release of therapeutic agents have been shown to enhance intra‐articular retention and protect cartilage by modulating key signaling pathways [34]. Beyond this, multifunctional and stimuli‐responsive hydrogels have been developed to inhibit angiogenesis and neurovascularization, thereby alleviating OA progression and associated pain [35]. Despite their advantages in drug delivery, hydrogel systems generally lack intrinsic antioxidative capacity, limiting their ability to directly modulate the oxidative microenvironment in OA.

To address this limitation, nanocatalysts with enzyme‐like activities, often referred to as nanozymes, have emerged as promising candidates due to their potent ROS‐scavenging functions in inflammatory disease models. Prussian blue nanozymes (PBNPs), ceria nanoparticles, and other metal‐based nanozymes not only neutralize ROS but also modulate macrophage polarization and mitochondrial homeostasis, thereby ameliorating local oxidative stress in arthritis and cartilage injury models [36, 37, 38, 39, 40, 41]. Emerging evidence further highlights their therapeutic versatility. Copper‐based metal‐organic framework nanozymes with multi‐enzyme‐like activities can efficiently eliminate diverse ROS species while suppressing pro‐inflammatory macrophage polarization and cartilage degradation [42]. Along this direction, biomimetic nanozymes have been engineered to integrate antioxidative activity with additional functionalities, such as lubrication and chondrogenic stimulation, enabling comprehensive restoration of joint homeostasis [43]. At a more mechanistic level, advanced dual‐site nanozymes have demonstrated enhanced ROS‐scavenging efficiency and mitochondrial protection, thereby inhibiting chondrocyte apoptosis and downstream inflammatory signaling during OA progression [44].

However, the spatiotemporally coordinated integration of miRNA therapeutics with nanozyme‐mediated redox regulation within a single responsive delivery platform remains largely unexplored in OA treatment.

**SCHEME 1:**
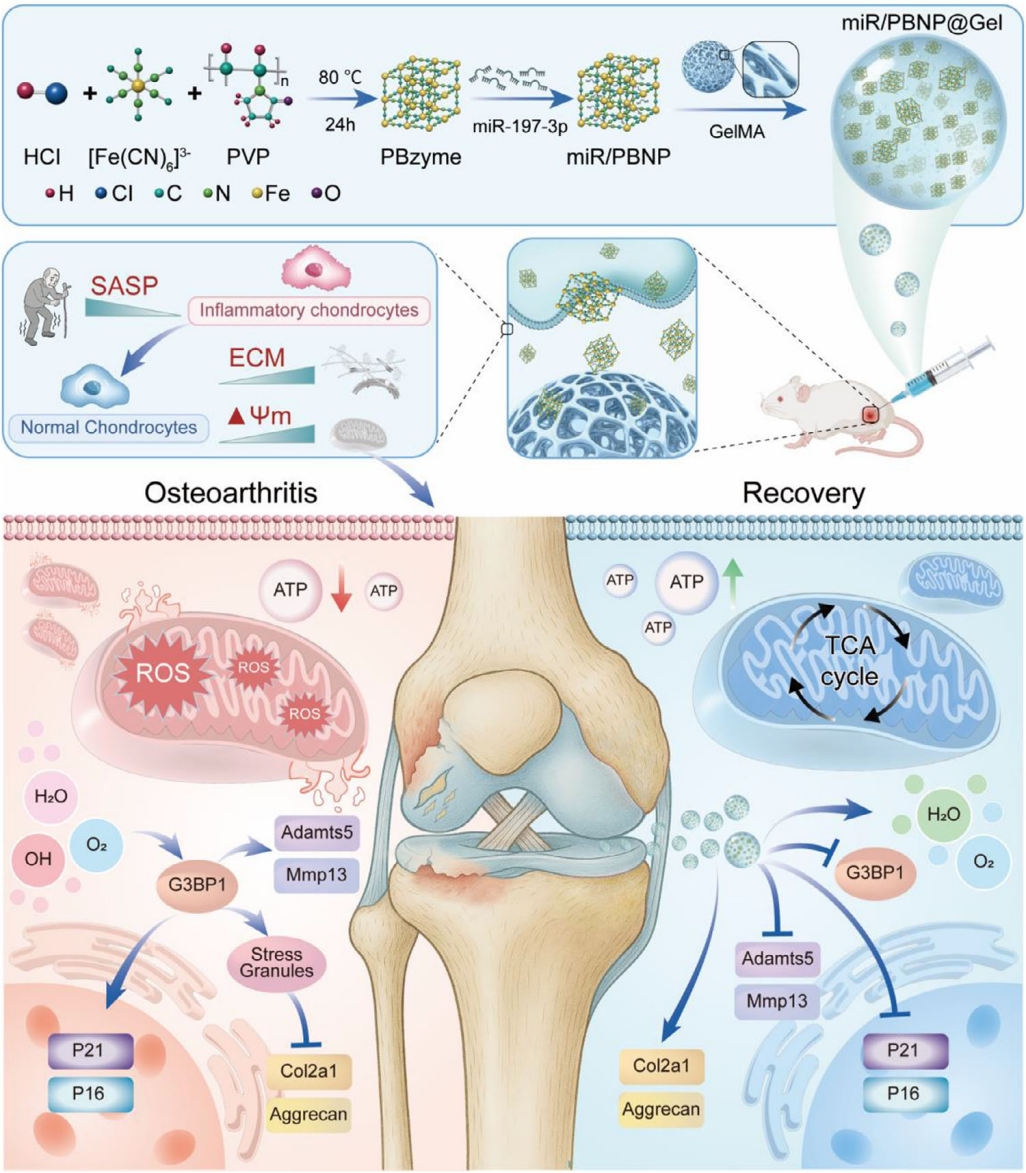
The schematic illustrated a multifunctional hydrogel platform (miR/PBNP@Gel) that co‐delivers miR‐197‐3p and PBNPs to modulate oxidative stress, reverse chondrocyte senescence, and restore cartilage function. PBNPs, Prussian blue nanozymes.

Here, we present a composite hydrogel microsphere platform co‐delivering miR‐197‐3p and PBNPs (miR/PBNP@Gel) for synergistic OA intervention. This integrated system enables efficient protection and sustained intra‐articular retention of miR‐197‐3p, enhanced cellular uptake, and simultaneous restoration of mitochondrial redox homeostasis through continuous ROS scavenging. Both in vitro and in vivo results demonstrate that this platform effectively suppresses chondrocyte senescence, promotes ECM anabolism, and reprograms the joint microenvironment (Scheme 1). Collectively, this work not only identifies miR‐197‐3p as a previously unrecognized therapeutic target for OA but also establishes a multifunctional delivery strategy for minimally invasive and translational cartilage regeneration.

## 2 Results

### 2.1 miR‐197‐3p Modulated Chondrocyte Senescence and Inflammation in OA Cartilage

**FIGURE 1:**
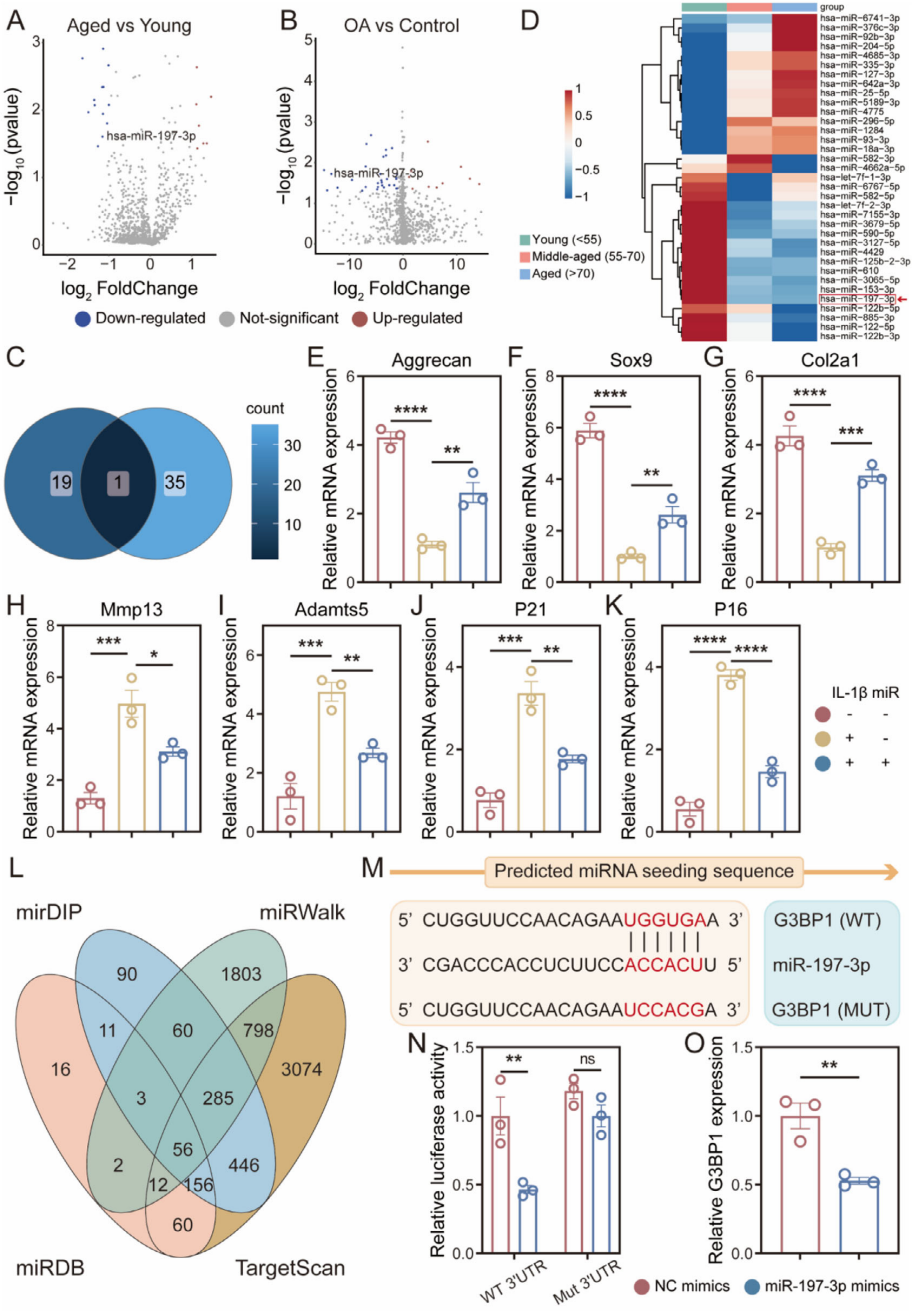
miR‐197‐3p alleviated chondrocyte senescence and inflammation in OA mouse articular cartilage. (A) Volcano plot of GSE222979 showing downregulation of miR‐197‐3p in aged cartilage. (B) Volcano plot of GSE143514 showing downregulation of miR‐197‐3p in OA cartilage. (C) Venn diagram indicating overlap of miR‐197‐3p downregulation across both datasets. (D) Heatmap of miR‐197‐3p expression across different age groups of OA patients. (E–K) qRT‐PCR analysis of inflammatory and senescence‐related gene expression in mouse chondrocytes treated with IL‐1β (10 ng/mL, 24 h) and transfected with miR‐197‐3p mimics (*n* = 3). qRT‐PCR, quantitative reverse transcription polymerase chain reaction. IL‐1β, interleukin‐1β. (L) Venn diagram of predicted miR‐197‐3p downstream target genes from multiple databases. (M) Schematic of unique miR‐197‐3p binding site within the 3’ UTR of G3BP1 mRNA. (N) Luciferase reporter assay of G3BP1 wild‐type and mutant constructs following transfection with miR‐197‐3p or NC mimics (*n* = 3). (O) qRT‐PCR analysis of G3BP1 mRNA levels following miR‐197‐3p transfection (*n* = 3). Data are presented as mean ± SEM. Two‐tailed Student's t‐test and one‐way ANOVA with Tukey's post hoc test was used for comparisons. *P < 0.05, **P < 0.01, ***P < 0.001, **** *P* < 0.0001. ns, not significant.

To explore the potential role of microRNAs in chondrocyte aging and OA pathogenesis, we conducted an integrated analysis of two transcriptomic datasets, GSE222979 and GSE143514, which include gene expression profiles of articular chondrocytes from different age groups and OA patients, respectively. Volcano plot analysis revealed that miR‐197‐3p was consistently downregulated in both datasets (Figure 1A–C). Moreover, z‐score‐normalized heatmap visualization across different age groups revealed a clear age‐associated pattern of miR‐197‐3p expression. Specifically, samples were stratified into young (<55 years), middle‐aged (55–70 years), and aged (>70 years) cohorts, showing a gradual decrease in miR‐197‐3p expression with advancing age (Figure 1D). This trend implies a potential association between reduced miR‐197‐3p expression and cartilage aging or OA‐related changes.

To further investigate the functional relevance of miR‐197‐3p in OA, we established an in vitro OA chondrocyte model by treating primary mouse chondrocytes with interleukin‐1β (IL‐1β, 10 ng/mL) for 24 h to mimic the inflammatory environment of OA. Quantitative reverse transcription polymerase chain reaction (qRT‐PCR) analysis showed that overexpression of miR‐197‐3p significantly upregulated the expression of key ECM synthesis genes, including Aggrecan, Sox9, and Col2a1, while downregulating matrix‐degrading enzymes and senescence‐associated genes such as Mmp13, Adamts5, P21, and P16 (Figure 1E–K). These results indicated that miR‐197‐3p promotes ECM homeostasis and suppresses chondrocyte catabolism and senescence under inflammatory conditions.

To elucidate the molecular targets of miR‐197‐3p, we performed in silico target prediction using multiple databases (miRWalk, mirDIP, miRDB, and TargetScan) and identified a set of putative downstream genes (Figure 1L). Among these, Ras GTPase‐activating protein‐binding protein 1 (G3BP1) emerged as a prominent target. G3BP1 is a key regulator of stress responses and stress granule (SG) assembly, processes closely linked to ROS signaling and cellular senescence. TargetScan Human 8.0 analysis revealed a complementary binding site between the miR‐197‐3p seed sequence and the 3’ untranslated region (3’UTR) of G3BP1 mRNA (Figure 1M). To validate this interaction, we constructed dual‐luciferase reporter vectors containing either the wild‐type (WT) or mutant (MUT) 3’UTR of G3BP1. As expected, luciferase activity was significantly reduced in the WT reporter upon miR‐197‐3p mimic transfection, whereas no significant change was observed with the MUT construct (Figure 1N). Furthermore, qRT‐PCR analysis confirmed that miR‐197‐3p mimic treatment significantly decreased G3BP1 mRNA levels in chondrocytes (Figure 1O). To establish G3BP1 as a functional mechanistic mediator of miR‐197‐3p, we performed complementary rescue and loss‐of‐function experiments. In IL‐1β‐treated chondrocytes, overexpression of G3BP1 (3’UTR‐deficient) partially reversed the protective effects of miR‐197‐3p on ECM synthesis, matrix‐degrading enzymes, and senescence markers, whereas G3BP1 knockdown phenocopied the effects of miR‐197‐3p mimic (Figures S1 and S2). Moreover, immunofluorescence analysis revealed that IL‐1β stimulation induced the formation of cytoplasmic G3BP1‐positive puncta in chondrocytes, which are widely regarded as a hallmark of stress granule assembly. This puncta formation was reduced by miR‐197‐3p overexpression, while reintroduction of G3BP1 partially restored this effect (Figure S3). Collectively, these data support G3BP1 as a key functional mediator linking miR‐197‐3p to cellular stress responses, including stress granule associated processes, redox imbalance, and chondrocyte senescence in OA.

### 2.2 Construction and Characterization of PBNP@Gel With Enhanced Antioxidant Performance

**FIGURE 2:**
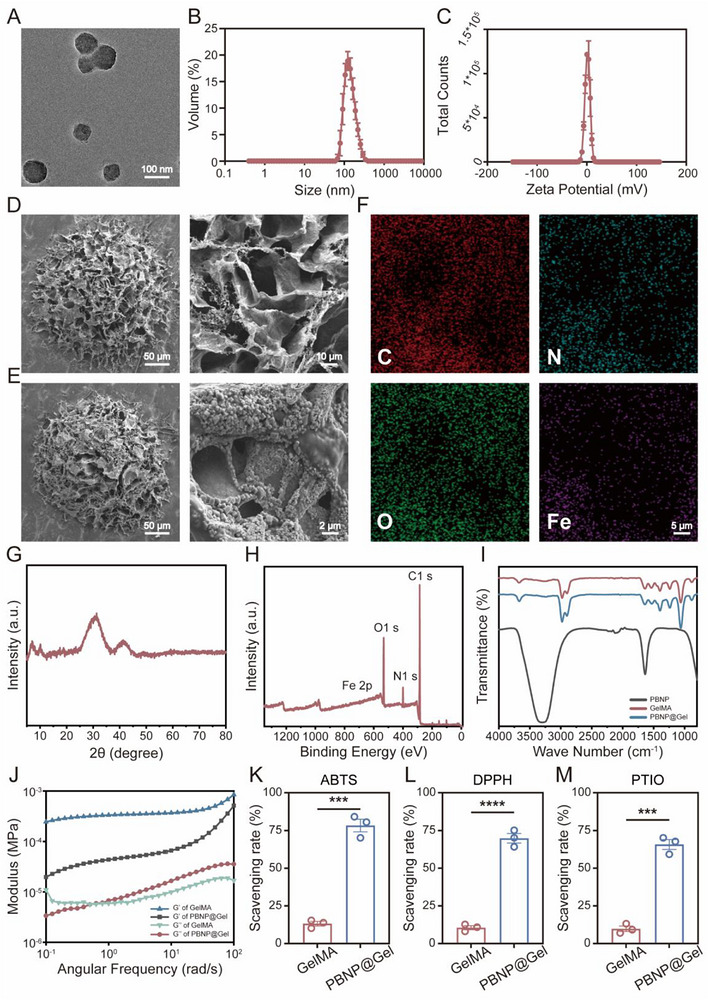
Construction and characterization of PBNP@Gel with enhanced antioxidant performance. (A) TEM image of PBNP (scale bar: 100 nm). TEM, transmission electron microscopy; PBNP, prussian blue nanozyme. (B, C) Size and zeta potential for PBNP, respectively. (D) SEM images of GelMA. Left: overview (scale bar: 50 µm); right: zoomed view (scale bar: 10 µm). SEM, scanning electron microscope. (E) SEM images of PBNP@Gel. Left: overview (scale bar: 50 µm); right: zoomed view (scale bar: 2 µm). (F) Energy dispersive spectroscopy results of PBNP@Gel (scale bar:5 µm), revealing the atomic distribution of C, O, N, and Fe. (G) X‐ray diffraction of PBNP. (H) X‐ray photoelectron spectroscopy of PBNP@Gel. (I) Fourier transform infrared spectroscopy of PBNP, GelMA, and PBNP@Gel. (J) Rheological measurements of storage (G') and loss (G'') moduli of GelMA and PBNP@Gel. (K‐M) Radical scavenging activity of GelMA and PBNP@Gel, including ABTS, DPPH, and PTIO radical scavenging assays (*n* = 3). Data are presented as mean ± SEM. Two‐tailed Student's t‐test was used for comparison. *** *P* < 0.001, **** *P* < 0.0001.

To construct a multifunctional therapeutic platform for intra‐articular miRNA delivery, PBNPs were encapsulated within a GelMA hydrogel matrix to form PBNP@Gel composites [45]. Transmission electron microscopy (TEM) revealed that nanozymes possesses a uniform cube morphology (Figure 2A). The particle size of pure PBNP was 152.4 ± 4.45 nm, with a polydispersity index (PDI) of 0.102 ± 0.065, indicating good monodispersity (Figure 2B). When PBNPs were incorporated into the GelMA hydrogel, the particle size significantly increased to 738.3 ± 27.05 nm and the PDI increased to 0.241 ± 0.048, suggesting that the nanoparticles aggregated or were encapsulated within the hydrogel (Figure S4). Additionally, the Zeta potential of pure PBNPs was slightly positive (1.112 ± 0.581 mV), indicating moderate stability (Figure 2C). In contrast, in the GelMA hydrogel, the Zeta potential of PBNP shifted to a negative value (‐10.51 ± 0.641 mV), significantly enhancing colloidal stability and reducing particle aggregation tendencies (Figure S5). These results suggested that the GelMA hydrogel alters the surface charge of the nanoparticles, increases their size, and may enhance their stability through the crosslinked network structure formed by GelMA.

Scanning electron microscope (SEM) images revealed interconnected porous networks of GelMA that are ideal for nutrient exchange and sustained release (Figure 2D). In the PBNP@Gel, PBNPs are densely distributed in the pores of the hydrogel (Figure 2E). Notably, energy dispersive spectroscopy elemental mapping confirmed homogeneous dispersion of Fe across the gel matrix, which is critical for ensuring consistent catalytic behavior throughout the scaffold (Figure 2F). The x‐ray diffraction (XRD) pattern of the original PBNP showed weak diffraction peaks at 2θ ≈ 7.27° and 10.23°, indicating some ordered structure, with broad peaks typical of Bi‐PBA's amorphous/nanocrystalline characteristics (Figure 2G). After crosslinking with GelMA, the XRD pattern revealed sharp diffraction peaks, suggesting a reorganization of the structure and enhanced long‐range order (Figure S6). The x‐ray photoelectron spectroscopy (XPS) analysis of PBNP@Gel revealed the characteristic peaks corresponding to the expected elements (Figure 2H).

Fourier transform infrared spectroscopy (FTIR) spectra of PBNP, GelMA, and PBNP@Gel showed no significant new peaks, indicating that the functional groups of the components were largely preserved throughout the incorporation process (Figure 2I). Importantly, rheological tests demonstrated that PBNP@Gel maintains desirable viscoelastic properties across physiological frequency ranges, with improved modulus over the pristine GelMA, indicating better mechanical robustness for intra‐articular administration (Figure 2J).

Functional assays showed that PBNP@Gel exhibited markedly enhanced scavenging efficiency across ABTS, DPPH, and PTIO assays (Figure 2K–M), attributable to the uniform distribution and surface availability of Fe active sites. Together, these results confirmed the successful fabrication of a biocompatible, catalytically active PBNP@Gel platform laying the foundation for its downstream biological applications in oxidative stress modulation and miRNA‐based cartilage repair. To further enable miRNA delivery, miR‐197‐3p was subsequently incorporated into the PBNP@Gel system by first forming miR/PBNP complexes, followed by encapsulation within the GelMA, yielding the miR/PBNP@Gel formulation used in the following experiments. Quantitative characterization of miR‐197‐3p encapsulation efficiency and loading content was performed for both miR@Gel and miR/PBNP@Gel, confirming effective miRNA incorporation (Figure S7). Furthermore, cumulative in vitro release studies in simulated synovial fluid (PBS containing 3 mg/mL BSA) over 14 days confirmed effective and sustained release of miR‐197‐3p (Figure S8). RNase stability assays also demonstrated that miR/PBNP@Gel effectively protected miR‐197‐3p from enzymatic degradation compared to free miRNA (Figure S9).

### 2.3 miR/PBNP@Gel Enhanced miR‐197‐3p Transfection Efficiency and Exhibited Excellent Biocompatibility in Chondrocytes

**FIGURE 3:**
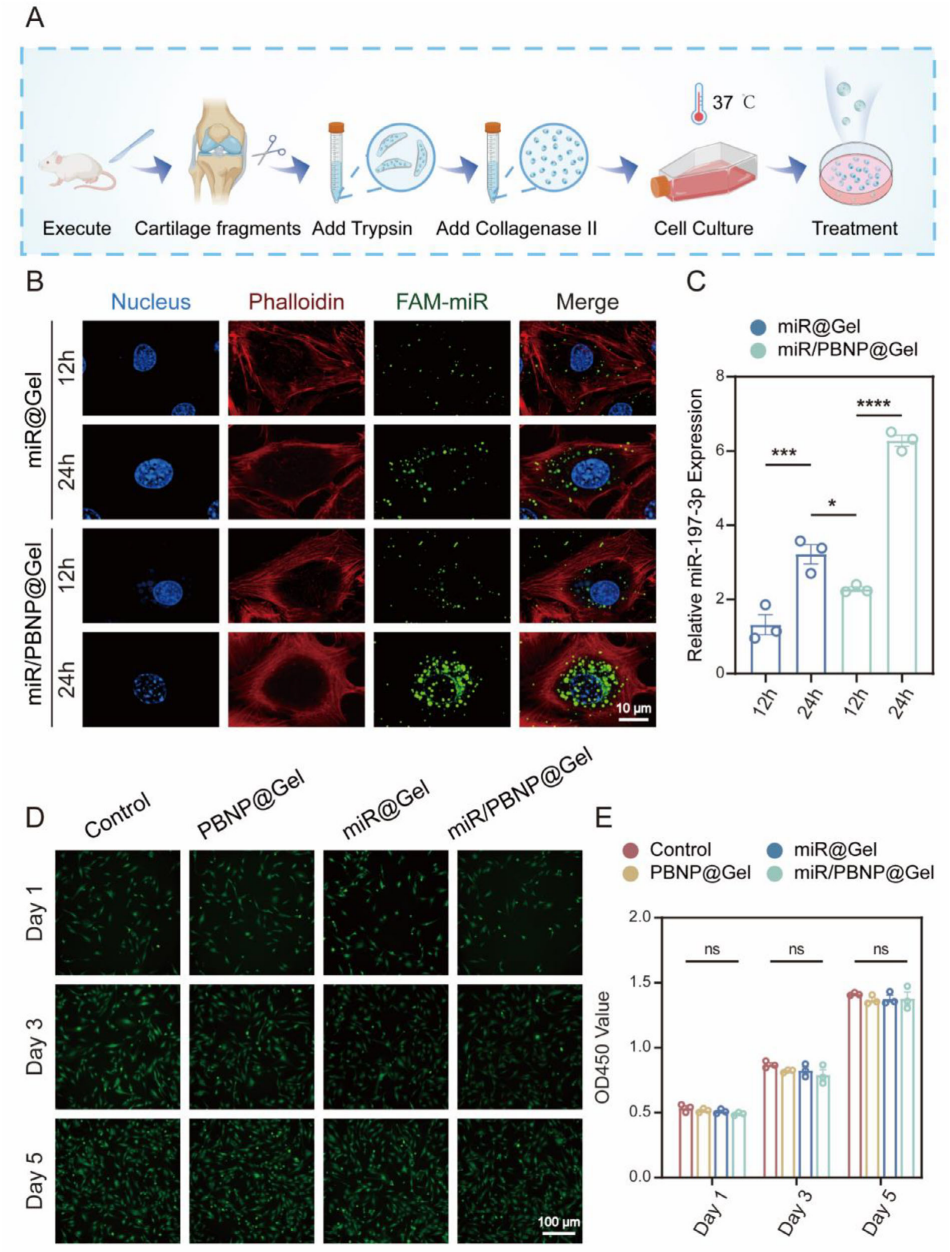
Transfection efficiency and in vitro biosafety evaluation of miR/PBNP@Gel. (A) Schematic illustration of chondrocyte isolation, culture, and co‐incubation with different materials. (B) CLSM images showing miR‐197‐3p expression in chondrocytes following transfection with miR@Gel or miR/PBNP@Gel (scale bar: 10 µm). CLSM, confocal laser scanning microscopy. (C) qRT‐PCR analysis of miR‐197‐3p expression in chondrocytes (*n* = 3). (D) CLSM images of live/dead staining of chondrocytes co‐cultured with materials on days 1, 3, and 5 (scale bar: 100 µm). (E) CCK‐8 assay of chondrocyte proliferation following co‐culture with different materials (*n* = 3). Data are presented as mean ± SEM. One‐way ANOVA with Tukey's post hoc test was used for multiple comparisons. * *P* < 0.05, ***P < 0.001, ****P < 0.0001, ns, not significant.

To assess the transfection efficiency and biocompatibility of the miR/PBNP@Gel platform in chondrocytes, we isolated and cultured primary mouse articular chondrocytes and incubated them with either miR@Gel or miR/PBNP@Gel formulations (Figure 3A). Confocal laser scanning microscopy (CLSM) imaging revealed that FAM‐labeled miR‐197‐3p was successfully internalized by chondrocytes and distributed homogeneously in the cytoplasm. Notably, miR/PBNP@Gel treated cells exhibited markedly higher intracellular fluorescence intensity at both 12 and 24 h post‐incubation compared to the miR@Gel group (Figure 3B). These observations were further corroborated by qRT‐PCR analysis, which demonstrated significantly elevated levels of intracellular miR‐197‐3p in the miR/PBNP@Gel‐treated group (Figure 3C). These results suggested that the presence of PBNP enhances miR‐197‐3p uptake by facilitating endocytosis and intracellular accumulation. The enzyme‐like catalytic activity of the nanozymes may further promote membrane translocation and cellular retention of miR‐197‐3p, thereby improving its therapeutic potential.

Next, we evaluated the cytocompatibility of the different formulations using live/dead staining. CLSM imaging showed that chondrocytes cultured with either PBNP@Gel, miR@Gel or miR/PBNP@Gel remained predominantly viable over a 5‐day period, with minimal dead cell staining observed across all groups (Figure 3D). To quantitatively assess cell proliferation, cell counting kit‐8 (CCK‐8) assays were performed. Chondrocytes treated with both formulations exhibited robust proliferation with no significant growth inhibition, further confirming their excellent cytocompatibility (Figure 3E).

Collectively, these findings demonstrated that miR/PBNP@Gel not only facilitates efficient miR‐197‐3p delivery to chondrocytes but also exhibits excellent biocompatibility and low cytotoxicity, supporting its suitability for intra‐articular therapeutic applications.

### 2.4 miR/PBNP@Gel Preserved ECM Synthesis and Mitigated Oxidative Stress, Senescence and Apoptosis in OA Chondrocytes

**FIGURE 4:**
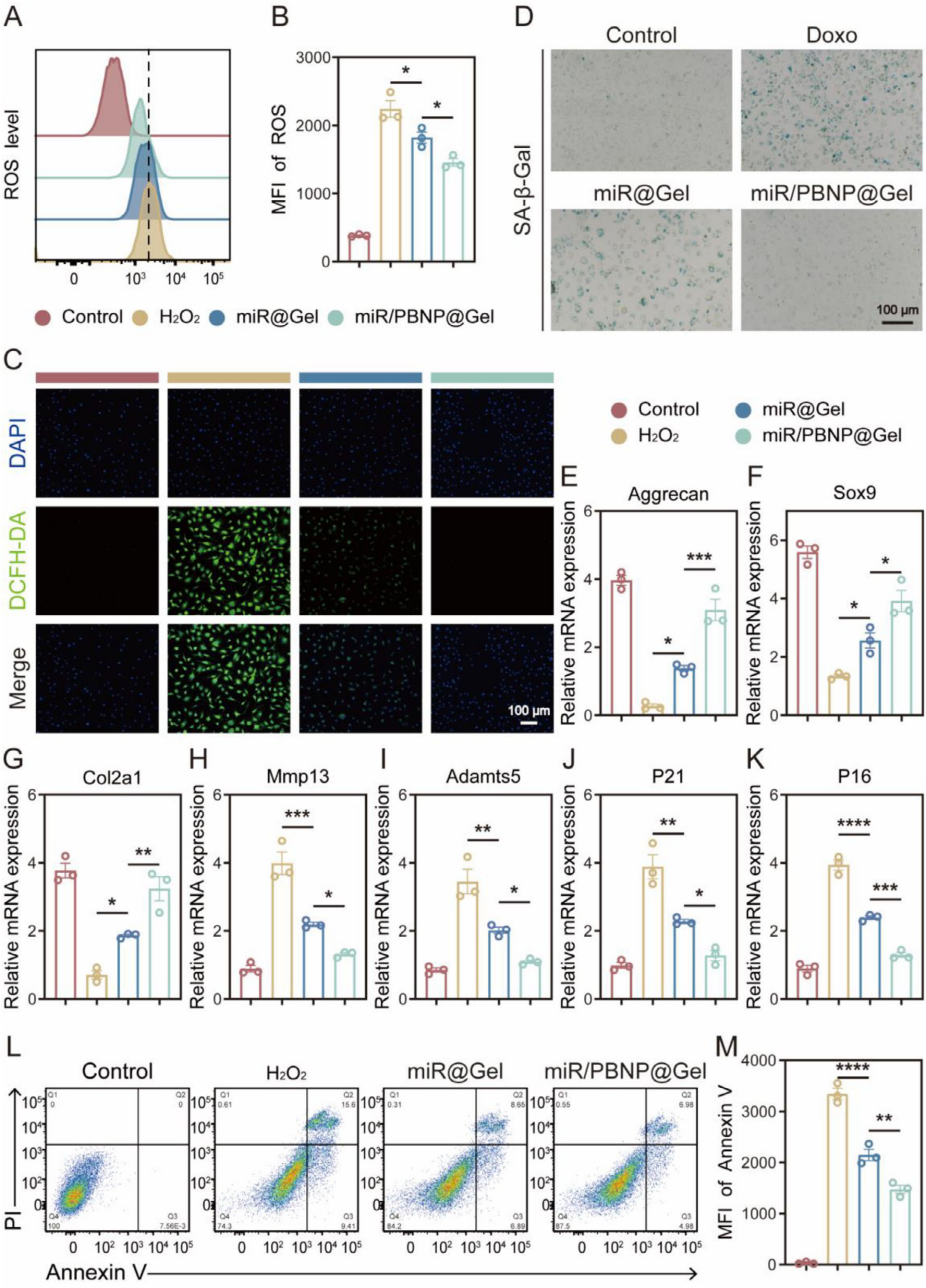
miR/PBNP@Gel alleviated oxidative stress, senescence, and inflammation in OA chondrocytes. (A) Flow cytometry analysis of ROS levels in chondrocytes treated with H_2_O_2_ (200 µM, 24 h) with different materials. H_2_O_2_, hydrogen peroxide. (B) Quantification of mean fluorescence intensity MFI of ROS staining (*n* = 3). (C) CLSM images of DCFH‐DA fluorescence intensity in chondrocytes treated with H_2_O_2_ (200 µM, 24 h) and different materials (scale bar: 100 µm). (D) SA‐β‐Gal staining of chondrocytes treated with Doxo (100 nM, 9 days) and co‐cultured with materials (scale bar: 100 µm). SA‐β‐Gal, senescence‐associated β‐galactosidase. Doxo, doxorubicin. (E–K) qRT‐PCR analysis of inflammatory and senescence‐related gene expression in chondrocytes treated with H_2_O_2_ (200 µM, 24 h) following different treatments (*n* = 3). (L, M) Flow cytometry analysis of apoptosis in chondrocytes treated with H_2_O_2_ (200 µM, 24 h) and different materials (*n* = 3). Data are presented as mean ± SEM. One‐way ANOVA with Tukey's post hoc test was used for multiple comparisons. *P < 0.05, **P < 0.01, ***P < 0.001, ****P < 0.0001.

ROS accumulation is a key contributor to chondrocyte damage and functional impairment in OA. Excessive ROS induces intracellular oxidative stress, disrupts cellular metabolism, and promotes chondrocyte death. To evaluate the antioxidative capacity of miR/PBNP@Gel, we measured intracellular ROS levels using the fluorescent probe 2’,7’‐dichlorodihydrofluorescein diacetate (DCFH‐DA). Flow cytometry analysis revealed that hydrogen peroxide(H_2_O_2_, 200 µM) stimulation for 24 h markedly elevated ROS production in chondrocytes, particularly at the mitochondrial level. Treatment with both miR@Gel and miR/PBNP@Gel significantly reduced ROS accumulation, with the miR/PBNP@Gel group exhibiting superior ROS‐scavenging efficacy (Figure 4A). Quantification of mean fluorescence intensity (MFI) further confirmed that ROS‐positive cell populations were significantly decreased following miR/PBNP@Gel treatment compared to H_2_O_2_ control (Figure 4B). The corresponding flow cytometry gating strategy and event distribution are provided in Figure S10. Consistent with these findings, CLSM imaging demonstrated visibly reduced green fluorescence intensity in the miR/PBNP@Gel group, indicating a robust antioxidative effect (Figure 4C). Supplementary experiments including GelMA and PBNP@Gel groups showed that PBNP@Gel alone partially reduced ROS levels, achieving a protective effect approaching that of miR@Gel, whereas miR/PBNP@Gel consistently produced the most pronounced ROS‐scavenging effect (Figure S11). The enhanced ROS clearance observed in the miR/PBNP@Gel group is likely attributable to the catalytic activity of the incorporated PBNPs.

In addition to ROS‐induced damage, accelerated chondrocyte senescence is a critical driver of OA progression, leading to impaired anabolic metabolism and ECM degradation. To evaluate the anti‐senescent effects of miR/PBNP@Gel, we established a chondrocyte senescence model by treating cells with doxorubicin (Doxo, 100 nM) for nine days. Senescence‐associated β‐galactosidase (SA‐β‐gal) staining revealed a significant increase in senescent cells in the Doxo treated group compared to the control. Both miR@Gel and miR/PBNP@Gel treatments reduced the proportion of SA‐β‐gal‐positive cells, with the miR/PBNP@Gel group showing the most pronounced reduction (Figure 4D). For completeness, additional groups including GelMA and PBNP@Gel were also analyzed, where PBNP@Gel also partially alleviated chondrocyte senescence (Figure S12). These results indicated that the nanozyme‐enhanced miRNA delivery platform effectively suppresses chondrocyte senescence.

Furthermore, qRT‐PCR analysis demonstrated that miR/PBNP@Gel treatment modulated the expression of key inflammation‐ and senescence‐related genes. Specifically, miR/PBNP@Gel upregulated expression of Aggrecan, Sox9 and Col2a1 while downregulating expression of Mmp13, Adamts5, P21 and P16 (Figure 4E–K). This gene expression profile is consistent with a shift toward a more regenerative and anti‐catabolic chondrocyte phenotype.

Apoptosis of chondrocytes is another pathological hallmark of OA, contributing to irreversible cartilage matrix loss. To assess the anti‐apoptotic effects of miR/PBNP@Gel, we performed Annexin V/PI dual staining and flow cytometry analysis. H_2_O_2_ exposure significantly increased the proportion of late apoptotic chondrocytes, whereas both miR@Gel and miR/PBNP@Gel treatments markedly reduced apoptosis rates (Figure 4L,M). The corresponding flow cytometry gating strategy and event distribution are provided in Figure S13. These findings further highlighted the protective effects of miR/PBNP@Gel on chondrocyte survival.

Collectively, these results demonstrated that miR/PBNP@Gel effectively mitigates oxidative stress, suppresses chondrocyte senescence and apoptosis, and restores ECM anabolic activity, thereby promoting the functional recovery of chondrocytes under OA‐mimicking conditions.

### 2.5 miR/PBNP@Gel Reshaped the Metabolic Network of OA Cartilage

To further investigate the regulatory effects of miR/PBNP@Gel on the metabolic state of OA cartilage, we performed untargeted metabolomics profiling on articular cartilage tissues from different treatment groups to assess its impact on metabolic network remodeling.

**FIGURE 5:**
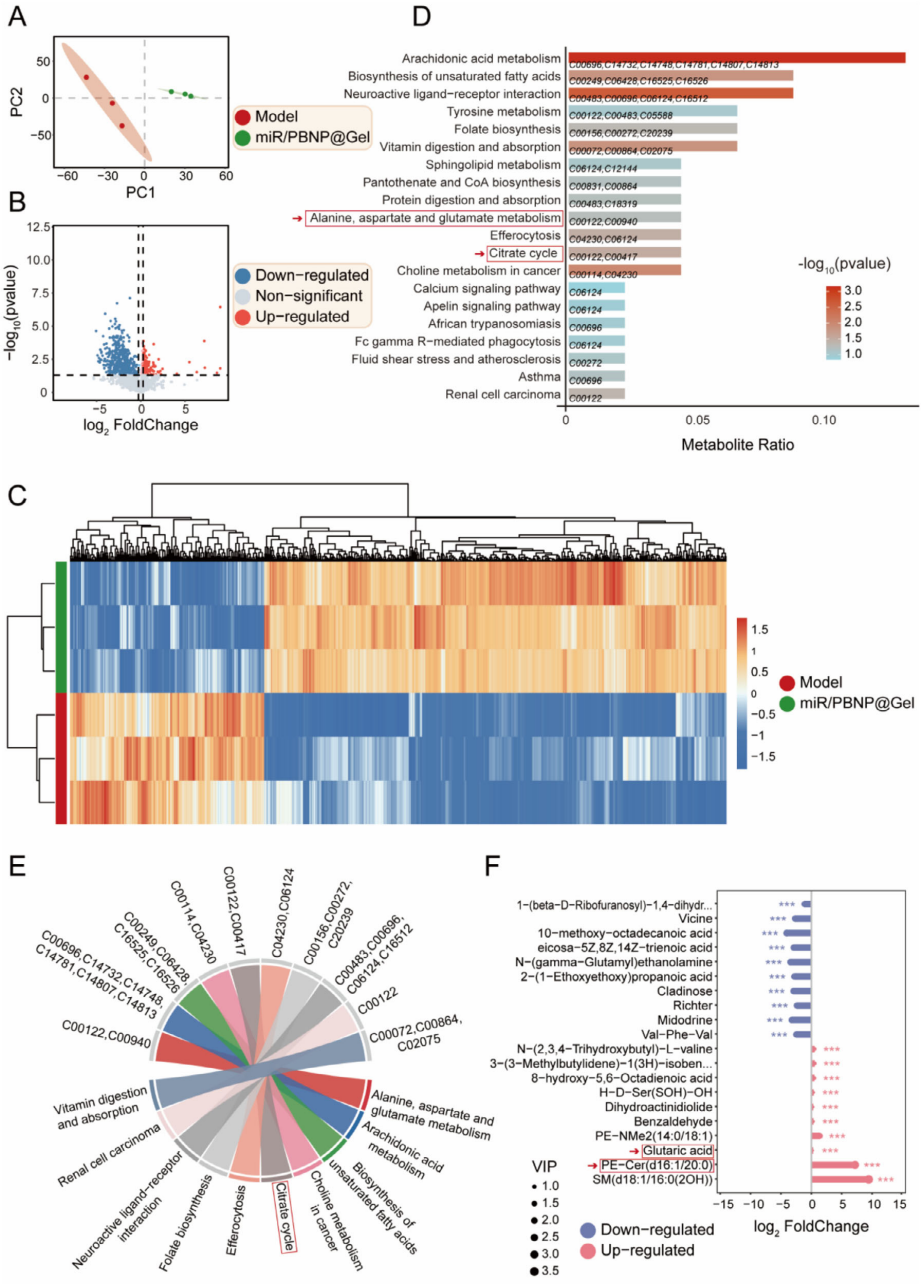
miR/PBNP@Gel remodeled the metabolic network of OA cartilage. (A) PCA of metabolomic profiles comparing Model and miR/PBNP@Gel groups. PCA, principal component analysis. (B) Volcano plot of upregulated (red) and downregulated (blue) differential metabolites. (C) Heatmap showing expression trends of differential metabolites across treatment groups. (D) KEGG pathway enrichment analysis of significantly enriched metabolic pathways. KEGG, encyclopedia of genes and genomes. (E) Pathway impact plot summarizing impact and contribution of differential pathways. (F) VIP score and expression trends of key differential metabolites, highlighting significantly upregulated or downregulated core metabolites. VIP, variable importance in the projection.

Principal component analysis (PCA) demonstrated a clear separation of metabolic profiles between the OA model (PBS) and miR/PBNP@Gel groups, indicating that miR/PBNP@Gel treatment was associated with substantial alterations in the metabolic profile of cartilage tissues (Figure 5A). Volcano plot analysis identified numerous metabolites that were significantly upregulated or downregulated in the miR/PBNP@Gel group, suggesting that the treatment broadly modulates cartilage metabolic homeostasis (Figure 5B). Heatmap visualization further revealed that the overall metabolic profile of the miR/PBNP@Gel group diverged from that of the OA model group, with several key metabolites exhibiting a trend toward restoration (Figure 5C). Kyoto Encyclopedia of Genes and Genomes (KEGG) pathway enrichment analysis showed that miR/PBNP@Gel treatment was associated with enrichment of multiple pathways related to energy metabolism and antioxidant processes, including glutamate, aspartate, and glutamine metabolism, tricarboxylic acid (TCA) cycle, pantothenate and coenzyme A biosynthesis, vitamin metabolism, and protein digestion and absorption pathways (Figure 5D,E). These findings suggest that, in addition to reducing oxidative stress, miR/PBNP@Gel may promote metabolic activities linked to energy production and biosynthesis, thereby potentially improving the functional state of chondrocytes.

Key differentially expressed metabolites included glutamate, aspartate, CoA‐related metabolites, lipid intermediates, and small‐molecule antioxidants, all of which exhibited high variable importance in the projection (VIP) scores and significant expression changes (Figure 5F and Table S1). The trends of these metabolic alterations closely paralleled the observed reductions in ROS and enhancement of ECM synthesis, further supporting the notion that miR/PBNP@Gel exerts multi‐layered regulatory effects on the OA cartilage microenvironment.

Together, these metabolomics findings indicate that miR/PBNP@Gel treatment is associated with a broad remodeling of the metabolic network of OA cartilage, characterized by coordinated changes in pathways related to antioxidant capacity, energy metabolism, and matrix biosynthesis, thereby contributing to its multifaceted biological efficacy in OA therapy.

### 2.6 miR/PBNP@Gel Restored Mitochondrial Function in OA Chondrocytes

Based on the findings from metabolomics sequencing, we further explored the changes in mitochondria. Mitochondrial homeostasis is crucial for maintaining chondrocyte function as the cell's energy powerhouse [46, 47]. In the context of OA, ROS and mitochondrial dysfunction exert interrelated roles. Inflammatory microenvironment stimulation or oxidative stress disrupts mitochondrial membrane potential, altering membrane permeability, which results in the release of proteins from the mitochondrial matrix into the cytoplasm. Furthermore, a decrease in membrane potential is also a hallmark of early apoptosis [48].

**FIGURE 6:**
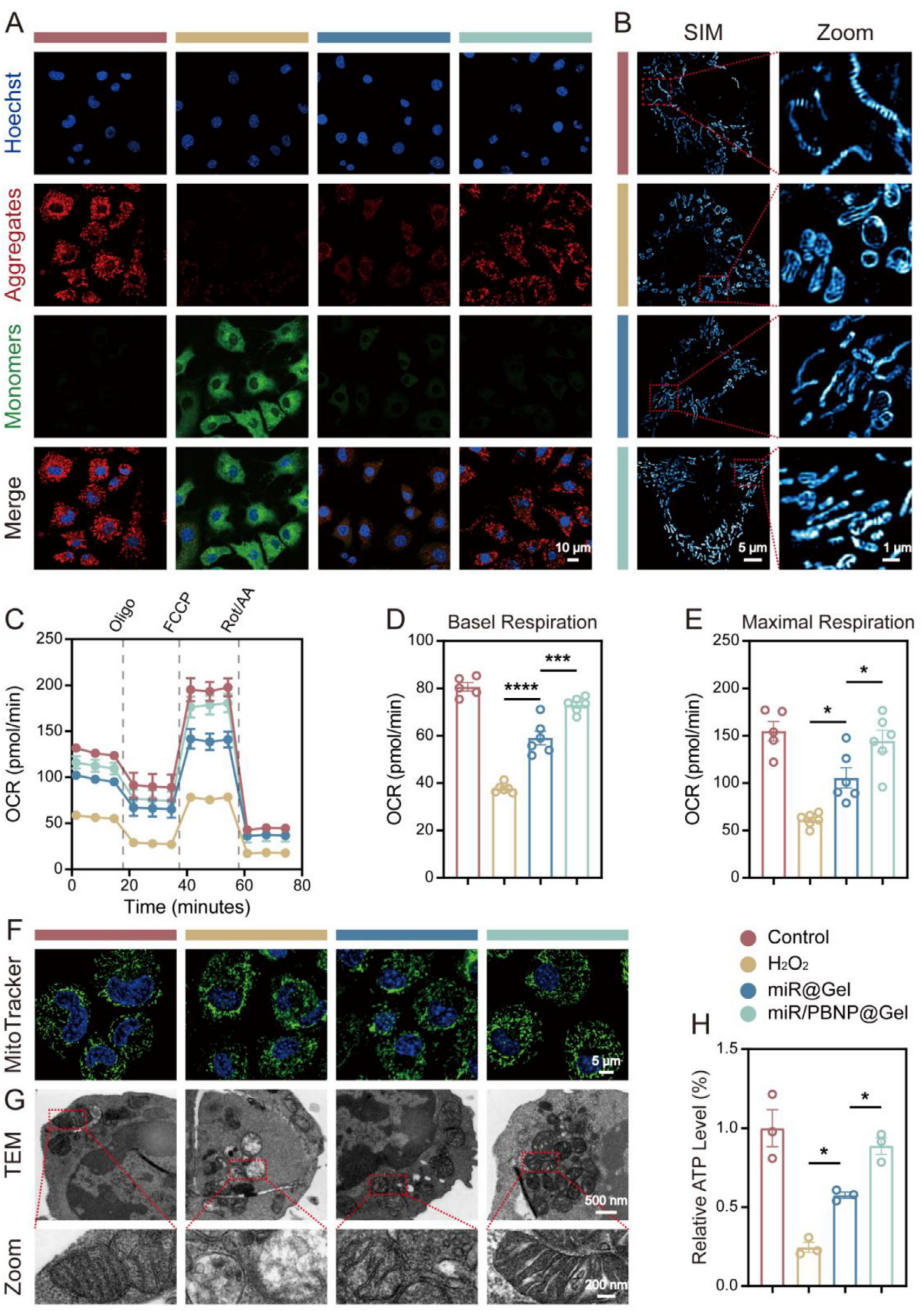
miR/PBNP@Gel restored mitochondrial function in OA chondrocytes. (A) JC‐1 staining of mitochondrial membrane potential in chondrocytes under H_2_O_2_ (200 µM, 24 h) and different treatments. Red indicates JC‐1 aggregates and green indicates JC‐1 monomers (scale bar: 10 µm). (B) SIM images mitochondrial cristae morphology in chondrocytes treated with H_2_O_2_ (200 µM, 24 h) with different materials (scale bar: 5 µm), and zoom images highlight cristae structure (scale bar: 1 µm). (C) Seahorse analysis of oxygen consumption rate over time in chondrocytes under H_2_O_2_ (200 µM, 24 h) and different treatments. (D‐E) Quantitative analysis of basal respiration and maximal respiration (*n* = 6). (F) MitoTracker Green staining of mitochondrial morphology in chondrocytes treated with H_2_O_2_ (200 µM, 24 h) with different materials (scale bar: 5 µm). (G) TEM imaging of chondrocyte mitochondrial ultrastructure following H_2_O_2_ (200 µM, 24 h) with different materials (scale bar: 500 nm), and zoom images highlight key structural details (scale bar: 200 nm). (H) Quantification of intracellular ATP content in chondrocytes treated with H_2_O_2_ (200 µM, 24 h) with different materials (*n* = 3). Data are presented as mean ± SEM. One‐way ANOVA with Tukey's post hoc test was used for multiple comparisons. *P < 0.05, ***P < 0.001, ***P < 0.0001.

Here, we employed the JC‐1 fluorescent probe to monitor the mitochondrial membrane potential under different treatments. Under normal conditions, JC‐1 aggregates in the mitochondrial matrix, emitting red fluorescence. When mitochondria are damaged, the membrane potential decreases, and JC‐1 exists solely in its monomeric form in the cytoplasm, emitting green fluorescence. CLSM images revealed that H_2_O_2_ stimulation notably disrupted the mitochondrial membrane potential, while miR@Gel treatment improved the membrane potential, and miR/PBNP@Gel treatment almost restored it to normal (Figure 6A). Additionally, PBNP@Gel exhibited a protective effect in restoring mitochondrial membrane potential that was slightly less pronounced than that of miR@Gel, as shown in Figure S14. Next, we used structured illumination microscopy (SIM) to visualize mitochondrial cristae morphology. Mitochondria were brightly fluorescent when stained with PK Mito Deep Red, and the cristae structure was clearly visible. In the H_2_O_2_ treated group, mitochondria exhibited swelling, vacuolization, and even rupture while miR/PBNP@Gel preserved the typical elongated and organized cristae structure, similar to control cells. These findings indicated that miR/PBNP@Gel protects mitochondrial ultrastructure from oxidative injury (Figure 6B).

To further investigate mitochondrial respiratory function, we performed Seahorse XF assays to monitor oxygen consumption rate (OCR) (Figure S15). Extracellular flux analysis revealed that H_2_O_2_ exposure significantly reduced both basal respiration and maximal respiratory capacity n chondrocytes. miR@Gel modestly improved respiration, while miR/PBNP@Gel significantly enhanced both basal and maximal OCR, restoring values close to those of control cells (Figure 6C–E). Notably, miR/PBNP@Gel also increased the spare respiratory capacity, indicating an improved ability of mitochondria to respond to energetic stress, and enhanced ATP‐linked respiration, reflecting more efficient coupling between oxidative phosphorylation and ATP production. Correspondingly, total isocitrate dehydrogenase (IDH) activity, a key enzyme in the TCA cycle, was significantly increased following miR/PBNP@Gel treatment, indicating an overall enhancement in TCA cycle‐associated metabolic activity (Figure S16). Consistent with these results, MitoTracker Green staining showed that H_2_O_2_ disrupted mitochondrial morphology, causing perinuclear clustering and fragmentation, whereas miR/PBNP@Gel treatment restored the normal filamentous mitochondrial network (Figure 6F). TEM further confirmed these protective effects, showing that control mitochondria displayed intact cristae and dense matrix, while H_2_O_2_ treatment induced vacuolization and cristae loss. miR/PBNP@Gel treatment preserved mitochondrial integrity and cristae architecture, which was further supported by semi‐quantitative analysis of cristae number (Figure 6G and Figure S17).

Finally, intracellular ATP content was found significantly decreased in H_2_O_2_ treated chondrocytes, but miR/PBNP@Gel treatment markedly restored ATP production (Figure 6H). These results demonstrated that miR/PBNP@Gel synergistically rescues mitochondrial membrane potential, morphology, respiration, and energy metabolism in OA chondrocytes.

### 2.7 miR/PBNP@Gel Improved Gait Function and Promoted Subchondral Bone Remodeling in OA Rats

**FIGURE 7:**
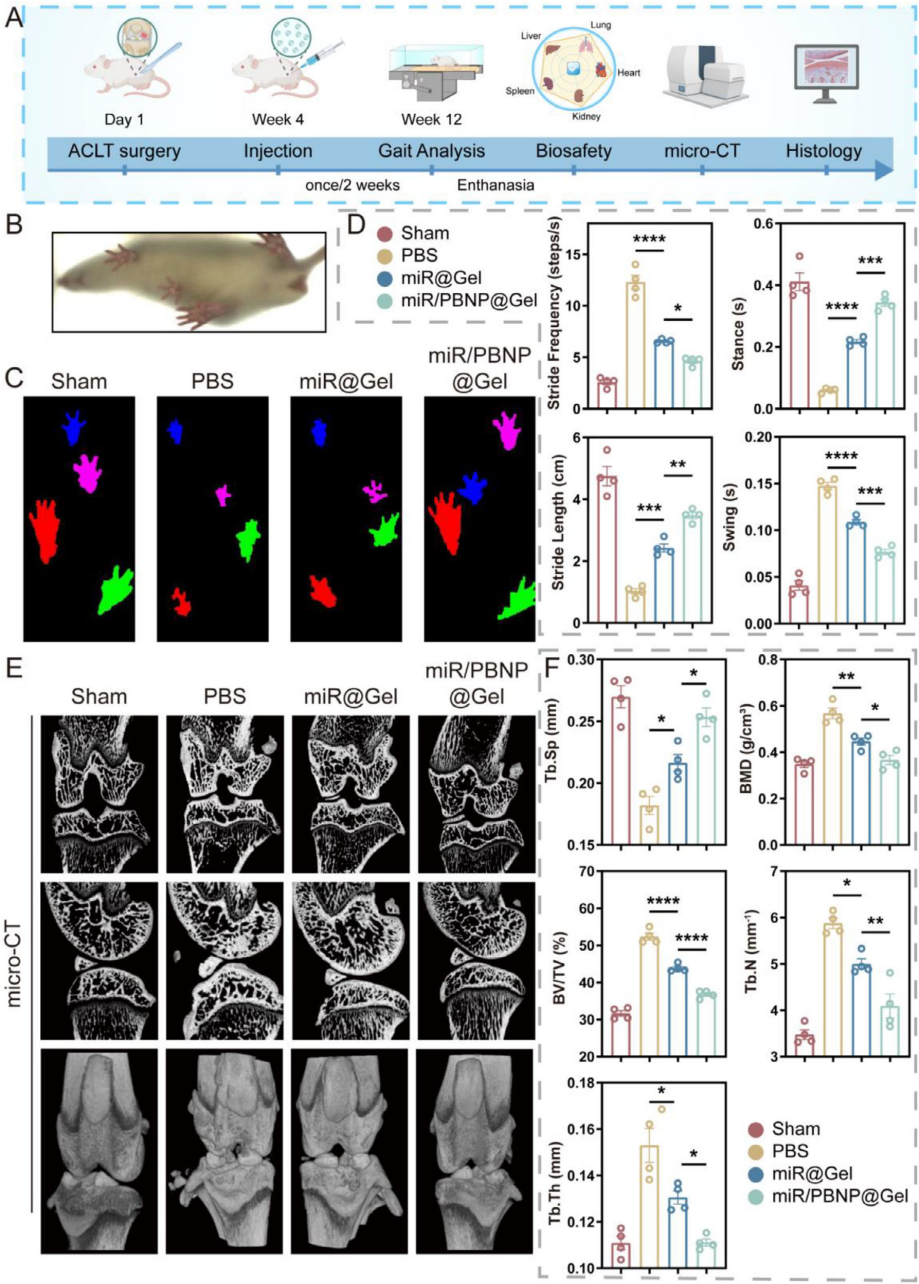
miR/PBNP@Gel improved gait function and promoted subchondral bone remodeling in OA rats. (A) Schematic illustration of experimental workflow including ACLT model induction, intra‐articular injection, gait analysis, and therapeutic assessment. (B) The ventral view of rats was recorded while walking on a transparent walking belt. (C) Digital paw prints of different groups generated by ventral plane imaging technology. (D) Quantitative analysis of gait parameters including stride frequency, stance time, stride length, and swing time (*n* = 4). (E) Micro‐CT imaging of subchondral bone microarchitecture in rat knees. (F) Quantitative analysis of subchondral bone parameters including Tb.Sp (trabecular separation), BMD (bone mineral density), BV/TV (bone volume fraction), Tb.N (trabecular number), and Tb.Th (trabecular thickness) (*n* = 4). Data are presented as mean ± SEM. One‐way ANOVA with Tukey's post hoc test was used for multiple comparisons. *p < 0.05, **p < 0.01, ***p < 0.001, ****p < 0.0001.

To assess the in vivo therapeutic effects of miR/PBNP@Gel, we used a rat model of OA induced by anterior cruciate ligament transection (ACLT), which is characterized by high reliability and reproducibility. Joint cavity injection was performed for four weeks post‐surgery, with treatments administered once every two weeks. The sham and control groups were treated with PBS at the same time. After eight weeks, rats were euthanized, and the knee joints were harvested (Figure 7A). To evaluate potential systemic toxicity, hematological and serum biochemical parameters, as well as histopathological examination of major organs, were assessed. No significant tissue damage or abnormalities were observed following treatment, suggesting that miR/PBNP@Gel exhibits favorable systemic biosafety (Figure S18). In addition, to evaluate intra‐articular retention and stability of the delivered miRNAs, Cy5‐labeled miR‐197‐3p, miR@Gel, and miR/PBNP@Gel were injected into rat knee joints. IVIS imaging was performed at 1, 7, and 14 days post‐injection. The results showed rapid clearance of naked miR‐197‐3p, partial retention with miR@Gel, and markedly prolonged retention with miR/PBNP@Gel, indicating that the combination of GelMA microspheres and nanozyme enhances intra‐articular miRNA retention (Figure S19).

Before euthanasia, the locomotor activity of animals was assessed using the DigiGait imaging system, which allows rats to run at a constant speed on a transparent motorized treadmill. A camera positioned beneath the treadmill records the ventral images of animals during movement and the images are automatically analyzed to generate digital paw prints and dynamic gait signals (Figure 7B). Gait analysis revealed that the sham group exhibited clear and stable gait patterns, with well‐defined and complete footprints. In contrast, the PBS group displayed disrupted gait and impaired mobility, reflected in smaller and less distinct footprints. miR@Gel treatment partially improved gait, whereas miR/PBNP@Gel treatment restored gait patterns to near‐normal levels (Figure 7C). Quantitative gait parameter analysis showed that PBS‐treated rats exhibited reduced stride frequency, prolonged stance time, shortened stride length, and decreased swing time, consistent with joint dysfunction. miR@Gel partially corrected these abnormalities, while miR/PBNP@Gel treatment significantly improved all gait parameters, restoring them to near‐normal levels (Figure 7D). These results demonstrated that miR/PBNP@Gel markedly enhances joint function in OA rats.

Micro‐computed tomography (micro‐CT) imaging and analysis of subchondral bone architecture revealed that the PBS group displayed noticeable subchondral bone sclerosis, osteophyte formation, and joint space narrowing. miR@Gel treatment partially ameliorated subchondral bone sclerosis, whereas miR/PBNP@Gel treatment robustly restored subchondral bone architecture, with well‐organized trabeculae and preserved joint space (Figure 7E). Quantitative analysis showed that PBS‐treated rats exhibited reduced trabecular separation (Tb.Sp), increased bone mineral density (BMD), elevated bone volume fraction (BV/TV), higher trabecular number (Tb.N), and increased trabecular thickness (Tb.Th). miR@Gel treatment moderately improved these parameters, while miR/PBNP@Gel treatment significantly restored them to near‐normal levels (Figure 7F). These findings indicated that miR/PBNP@Gel not only promotes cartilage repair but also prevents OA‐associated subchondral bone hardening, thereby improving the bone‐cartilage interface microenvironment and enhancing overall joint mobility.

### 2.8 miR/PBNP@Gel Attenuated Chondrocyte Senescence and Restored Cartilage Matrix Metabolism in Vivo

**FIGURE 8:**
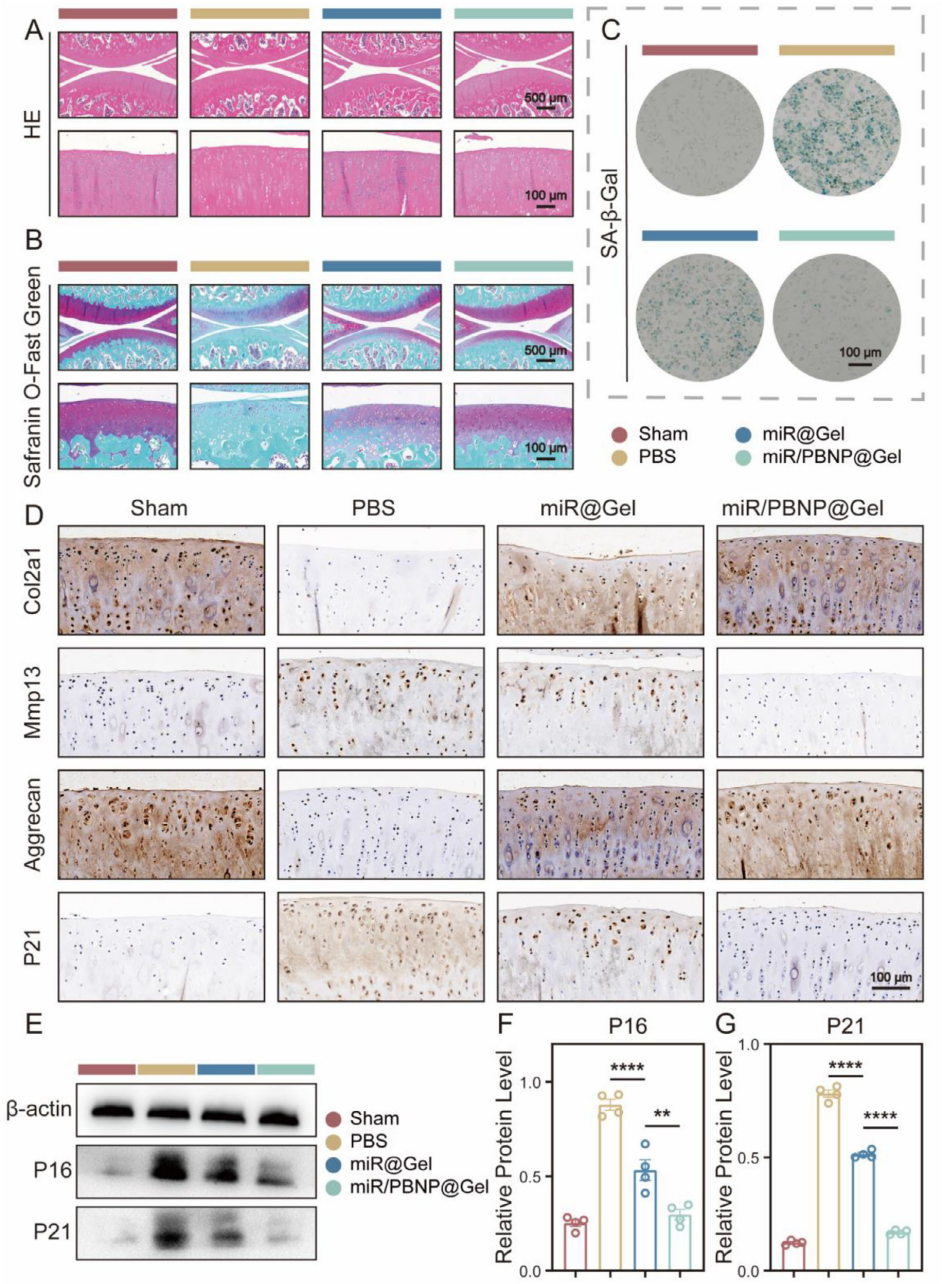
miR/PBNP@Gel attenuated chondrocyte senescence and improved cartilage matrix metabolism. (A) Hematoxylin and eosin staining of cartilage tissue morphology in different treatment groups. Up: overview (scale bar: 500 µm); Down: zoomed view (scale bar: 100 µm). (B) Safranin O/fast green staining of cartilage glycosaminoglycan content. Up: overview (scale bar: 500 µm); Down: zoomed view (scale bar: 100 µm). (C) SA‐β‐Gal staining of senescent chondrocytes in cartilage tissues (scale bar: 100 µm). (D) Immunohistochemical staining of Col2a1, Mmp13, Aggrecan and P21 in cartilage tissues (scale bar: 100 µm). (E‐G) Western blot analysis and grayscale quantification of P16 and P21 protein expression in cartilage tissues (*n* = 4). Data are presented as mean ± SEM. One‐way ANOVA with Tukey's post hoc test was used for multiple comparisons. **p < 0.01, ****p < 0.0001.

To further elucidate the in vivo effects of miR/PBNP@Gel on chondrocyte senescence and matrix metabolism, we conducted comprehensive histological, senescence staining, immunohistochemical, and protein‐level analyses of treated cartilage tissues. Hematoxylin and eosin (H&E) staining and safranin O/fast green (SO&FG) staining demonstrated that sham group cartilage exhibited intact structure and abundant glycosaminoglycan (GAG) content, whereas PBS‐treated cartilage showed severe surface erosion, disrupted architecture, and significant matrix loss. miR@Gel treatment partially improved cartilage structure and matrix integrity, while miR/PBNP@Gel treatment restored surface continuity and significantly enhanced GAG staining, indicating improved ECM synthetic capacity (Figure 8A,B).

We then isolated cartilage cells from rats in different treatment groups to assess cellular senescence. SA‐β‐Gal staining revealed a marked increase in senescent chondrocytes in the PBS group, which was reduced by miR@Gel treatment and most significantly suppressed in the miR/PBNP@Gel group (Figure 8C). Immunohistochemical analysis further showed that Col2a1 and Aggrecan expression was reduced in the PBS group and restored to near‐normal levels by miR/PBNP@Gel treatment, while Mmp13 and P21 expression was elevated in OA cartilage and significantly reduced following miR/PBNP@Gel treatment (Figure 8D).

To corroborate these findings, we performed western blot analysis of cartilage tissues. PBS‐treated cartilage exhibited elevated P16 and P21 protein levels, while miR@Gel treatment reduced their expression, and miR/PBNP@Gel treatment further suppressed these proteins, approaching the levels observed in the sham group (Figure 8E–G). These results, consistent with histological and immunohistochemical findings, confirmed that miR/PBNP@Gel effectively modulates chondrocyte senescence pathways, improves the OA cartilage microenvironment and promotes tissue repair.

Together, these in vivo data demonstrated that miR/PBNP@Gel not only improves cartilage structural integrity and matrix metabolism but also exerts potent anti‐senescent effects, thereby providing strong mechanistic support for its disease‐modifying efficacy in OA.

To further evaluate the therapeutic efficacy of different materials under aging‐associated osteoarthritic conditions, we additionally examined its effects in aged guinea pigs, a well‐established spontaneous model of age‐related osteoarthritis. Unlike surgically induced OA models, aged guinea pigs naturally develop progressive cartilage degeneration and joint structural changes with aging. Micro‐CT imaging together with histological analyses, including H&E and SO&FG staining, demonstrated that treatment with the materials markedly alleviated cartilage degeneration and improved joint structural integrity compared with untreated aged animals. These results further support the therapeutic potential of miR/PBNP@Gel in age‐related osteoarthritic conditions (Figure S20).

## 3 Discussion

OA is a complex degenerative disease characterized by the gradual degradation of articular cartilage, subchondral bone remodeling, and synovial inflammation. As the disease progresses, the function of chondrocytes deteriorates, leading to an imbalance in the ECM, exacerbating cartilage degradation. Despite significant advances in our understanding of the biological mechanisms underlying OA, current treatments remain primarily symptom‐based and do not effectively repair damaged cartilage or reverse the pathological processes. Common therapeutic strategies include pain relief, physical therapy, and surgical interventions, however, these approaches do not fundamentally alter the disease course. Pathogenesis of OA is closely linked to oxidative stress, chondrocyte senescence, and ECM remodeling dysregulation, creating an urgent need for disease‐modifying osteoarthritis drugs that can intervene in these pathological pathways.

Recent studies have supported the potential of miRNA‐based therapies for OA [49]. For instance, Zhu et al. [50] developed a stem cell‐homing hydrogel delivering miR‐29b‐5p, which suppressed chondrocyte senescence and promoted cartilage regeneration. Similarly, Li et al. [51] designed a ZnO‐based hydrogel to deliver miR‐17‐5p, which enhanced ECM synthesis and reduced ECM degradation through sustained miRNA release and zinc ion‐mediated mesenchymal stem cell recruitment. These studies, along with our own work, highlight the potential of miRNA as a therapeutic strategy for OA. However, existing delivery systems face challenges such as rapid degradation and low cellular uptake, necessitating the development of innovative methods to enhance their stability and efficacy.

In this study, we presented an innovative multifunctional therapeutic platform that integrates PBNP‐based hydrogel microspheres with the delivery of miR‐197‐3p, targeting key drivers of OA progression, including oxidative stress, chondrocyte senescence and ECM dysregulation. Within this system, PBNPs exhibit a structurally derived dual functionality. The nanoscale framework provides abundant redox‐active sites that support catalytic ROS scavenging, while the surface coordination capability enables efficient complexation with miR‐197‐3p via coordination and electrostatic interactions, enhancing its stability against degradation and promoting intracellular delivery. Notably, these catalytic and delivery functions are functionally interconnected. Reduction of intracellular oxidative stress stabilizes the cellular environment and supports miRNA activity, whereas effective delivery of miR‐197‐3p enables regulation of stress‐responsive pathways. This coupling establishes a coordinated mechanism linking microenvironment modulation with gene regulation. Notably, a similar integrative strategy combining nanozyme‐mediated ROS scavenging with miRNA regulation has also been reported in inflammatory joint diseases such as rheumatoid arthritis, where synergistic modulation of oxidative stress and pro‐inflammatory signaling further supports the therapeutic potential of this dual‐functional approach [52].

Meanwhile, the use of GelMA microspheres offered additional advantages, including tunable degradation, enhanced injectability and sustained intra‐articular retention, enabling localized and prolonged therapeutic effects. This integrated design addresses key limitations of conventional miRNA delivery systems, particularly instability and low intracellular availability. As a result, overexpression of miR‐197‐3p in OA chondrocytes suppressed the secretion of SASP factors, upregulated anabolic ECM genes and downregulated catabolic enzymes. Furthermore, we identified G3BP1, a stress granule assembly factor, as a direct target of miR‐197‐3p. Importantly, complementary rescue and loss‐of‐function experiments further demonstrated that G3BP1 mediates the effects of miR‐197‐3p on chondrocyte function, including ECM metabolism and cellular senescence. In addition, modulation of miR‐197‐3p altered the formation of G3BP1‐positive puncta under stress conditions, suggesting the involvement of G3BP1‐associated cellular stress responses. Together, these results suggest that miR‐197‐3p contributes to the regulation of oxidative stress and chondrocyte homeostasis in OA, at least in part through targeting G3BP1.

In vivo studies demonstrated that miR/PBNP@Gel significantly slowed cartilage degeneration in rat OA models. Micro‐CT analysis revealed improvements in subchondral bone structure. Histological examination confirmed the preservation of cartilage matrix, with increased expression of anabolic ECM components and reduced markers of senescence and catabolism. Importantly, no systemic toxicity or adverse effects were observed, supporting the biocompatibility of our therapeutic platform.

Despite these encouraging outcomes, several limitations should be acknowledged. The long‐term fate of PBNPs within joint tissues and the potential cumulative effects associated with repeated intra‐articular administration remain to be systematically investigated. Although prior studies have suggested good biocompatibility of ultrasmall PBNPs, comprehensive evaluation in large‐animal models and under chronic exposure conditions will be essential to support clinical translation. In parallel, while miR‐197‐3p exhibited robust therapeutic efficacy, a more in‐depth assessment of its potential off‐target effects and long‐term safety profile is still required. Importantly, emerging theranostic platforms based on enzyme‐responsive molecular beacons activated by OA‐associated proteases, including MMP‐13, have demonstrated the potential for simultaneous disease imaging and targeted therapy, highlighting a promising direction for achieving more precise and personalized OA intervention [53]. In a broader context, advances in bioimaging materials, such as surface‐engineered persistent luminescence systems with improved stability and biocompatibility, further underscore the growing interest in integrating diagnostic and therapeutic functionalities for biomedical applications [54].

## 4 Conclusion

In conclusion, we introduced a multifunctional, minimally invasive therapeutic platform that integrates nanozyme‐based redox control, miRNA‐driven gene modulation, and biomaterial‐based delivery to address key pathophysiological drivers of OA. By restoring mitochondrial function, reducing oxidative stress and SASP signaling, and promoting cartilage matrix synthesis, our miR/PBNP@Gel platform represented a promising disease‐modifying strategy. As precision nanomedicine advances, such integrated approaches hold strong potential to transform OA management and enable new therapeutic avenues for degenerative joint diseases.

## 5 Materials and Methods

### 5.1 Study Design

This study aimed to evaluate the synergistic therapeutic potential of a miR‐197‐3p loaded nanozyme delivery platform for OA, with a focus on its ability to mitigate chondrocyte senescence, oxidative stress and ECM imbalance. We engineered an injectable, ROS‐scavenging delivery platform (miR/PBNP@Gel) by co‐encapsulating miR‐197‐3p and PBNPs within microfluidics‐fabricated GelMA hydrogel microspheres. Initially, miR‐197‐3p was identified as a therapeutically relevant miRNA due to its consistent downregulation in both aged and osteoarthritic cartilage. Subsequent in vitro functional assays demonstrated the therapeutic advantages of miR/PBNP@Gel in rescuing OA chondrocyte phenotypes. In vivo efficacy was evaluated through gait analysis, micro‐CT, histological staining, and immunohistochemistry. Rats were randomly assigned to treatment groups prior to intervention. No exclusion criteria were applied to any of the collected experimental data. All animal experiments were approved by the Animal Welfare and Ethics Committee of Peking Union Medical College Hospital (XHDW‐2023‐021) and conducted in accordance with institutional and national ethical guidelines for laboratory animal care.

### 5.2 Fabrication and Characterization of PBNP

Based on previous research methods for the preparation of Prussian blue nano particles, PVP (8.0 g) and K_3_[Fe(CN)_6_] (696 mg) were mixed with HCl solution (1 M, 50 mL). After stirring for 1 h, the solution was transferred to an electric oven and heated at 80°C for 24 h. Finally, PBNP was obtained by centrifugation and washed 5 times with deionized water. The morphology of PBNP was characterized using transmission electron microscopy (TEM; Carl Zeiss, Germany), and the particle size and zeta potential were measured using dynamic light scattering.

### 5.3 Fabrication of Mir/Pbnp

For in vitro experiments, miR‐197‐3p mimics were used to prepare miR/PBNP complexes and applied to cultured cells at a final miR‐197‐3p concentration of 50 nM. To prepare the complexes, miR‐197‐3p mimics and PBNP were both dissolved in diethylpyrocarbonate (DEPC)‐treated water and mixed together and gently vortexed. The mixture was incubated at room temperature with continuous shaking at 250 rpm for 30 min to ensure homogeneous mixing. The resulting miR/PBNP composite was collected by centrifugation at 10 000 rpm for 10 min, and the supernatant was discarded.

### 5.4 Fabrication of GelMA Hydrogel Microspheres and PBNP@Gel

Gelatin methacryloyl (GelMA) hydrogel microspheres were fabricated using a microfluidic approach. To prepare the photoinitiator solution, 0.25% (w/v) lithium phenyl (2,4,6‐trimethylbenzoyl) phosphinate (LAP) was dissolved in PBS and heated at 40°C–50°C for 15 min to ensure complete dissolution. GelMA was then added to the LAP solution to achieve a final concentration of 7% (w/v). The mixture was gently stirred at 45°C for 30–60 min to fully dissolve GelMA, followed by sterilization using a 0.22 µm filter. For microsphere generation, the GelMA solution (dispersed phase) and liquid paraffin containing 10% (w/v) span 80 (continuous phase) were loaded into separate syringes connected to a microfluidic droplet generator. Monodisperse droplets were formed at flow rates of 20 µL/min for the dispersed phase and 100 µL/min for the continuous phase. These droplets were subsequently exposed to ultraviolet light (405 nm, 10 mW/cm^2^) for photo‐crosslinking, resulting in stable GelMA hydrogel microspheres. The obtained microspheres were washed sequentially with PBS and 75% ethanol to remove residual oil and surfactants. Finally, the microspheres were immersed in PBS for 4 h, with four solvent exchanges performed to ensure complete washing and equilibration. PBNP were dispersed in PBS to form a homogeneous suspension, after which they were gently added to the GelMA microsphere suspension under continuous stirring. The mixture was stirred for 1 h at room temperature to ensure uniform distribution of PBNP within the hydrogel matrix. The resulting PBNP@Gel composite was then subjected to further sterilization and used for subsequent applications.

### 5.5 Rheological Testing

The rheological properties of Gel and PBNP@Gel hydrogels were characterized using a rotational rheometer. Prior to testing, the samples were carefully prepared to ensure smooth surfaces free of bubbles or impurities. Rheological measurements were performed at 25°C, with the angular frequency gradually decreasing from 100 to 0.1 rad/s while maintaining a constant strain of 5%. The viscoelastic behavior of the hydrogels was evaluated by analyzing the storage modulus (G‘) and loss modulus (G’).

### 5.6 TEM

The samples were fixed in 2.5% glutaraldehyde and rinsed with PBS. They were then post‐fixed in 1% osmium tetroxide. After graded ethanol dehydration and acetone exchange, specimens were embedded in Embed‐812 resin. Ultrathin sections (≈ 70 nm) were cut with an ultramicrotome and mounted on copper grids. Sections were stained with 2% uranyl acetate followed by lead citrate. Images were captured with a transmission electron microscope (JEM‐1400Plus, JEOL, Japan).

### 5.7 Scanning Electron Microscopy

Specimens were fixed in 2.5% glutaraldehyde and rinsed with PBS. They were dehydrated through a graded ethanol series and dried by critical point drying. Surfaces were sputter‐coated with a thin layer of gold. Samples were observed with a scanning electron microscope (SU8010, Hitachi, Japan) at an accelerating voltage of 5 kV. Additionally, energy‐dispersive x‐ray spectroscopy (EDS) was performed on the PBNP@Gel samples to analyze elemental composition.

### 5.8 Antioxidant Activity Detection

Reactive oxygen species (ROS) generation was assessed using Total Antioxidant Capacity Assay Kit with ABTS method (Beyotime, China, S0119), DPPH Free Radical Scavenging Capacity Assay Kit (Solarbio, China, BC4750), and PTIO Free Radical Scavenging Capacity Assay Kit (Aidisheng, China, ADS‐W‐KY027‐196). After incubating the samples for 48 h, absorbance changes were measured to reflect ROS neutralization. These measurements were used to quantify the ROS levels and the antioxidant capacity of the samples.

For the ABTS assay, 200 µL of ABTS working solution was mixed with 10 µL of sample or Trolox standard (0.15–1.5 mM) and incubated at room temperature for 6 min in the dark, after which absorbance was recorded at 734 nm. For the DPPH assay, 950 µL of DPPH working solution was mixed with 50 µL of sample or vitamin C solution (10 mg/mL) and incubated at room temperature for 30 min in the dark, followed by measurement of absorbance at 515 nm. For the PTIO assay, 100 µL of sample was mixed with 200 µL of PTIO working solution and incubated at 37°C for 30 min in the dark, after which absorbance was recorded at 557 nm. In all assays, radical scavenging activity was expressed as the percentage decrease in absorbance relative to the control after blank correction.

### 5.9 miRNA Encapsulation and Loading

The encapsulation efficiency (EE) and loading content (LC) of miR‐197‐3p were determined by measuring the amount of unencapsulated miRNA in the supernatant. Briefly, miR@Gel and miR/PBNP@Gel were centrifuged at 12 000 g for 10 min, and the supernatant was collected. The concentration of free miRNA was quantified by measuring absorbance at 260 nm using a NanoDrop spectrophotometer. EE and LC were calculated according to the following equations:

$$\mathrm{EE}\;\left(\%\right)=\frac{W_{\mathrm{total}}-W_{\mathrm{free}}}{W_{\mathrm{total}}}\;\times 100\%$$

$$\mathrm{LC}\;\left(\%\right)=\frac{W_{\mathrm{encapsulated}}}{W_{\mathrm{carrier}}}\;\times 100\%$$

where Wtotal is the initial amount of miRNA added, Wfree is the amount of unencapsulated miRNA measured in the supernatant, Wencapsulated is the amount of miRNA retained in the hydrogel, and Wcarrier is the total mass of the hydrogel carrier.

### 5.10 Detection of mRNA Expression

Total RNA was extracted from cells with TRIzol Reagent (Invitrogen, USA, 15596018CN) according to the manufacturer's instructions. One microgram of RNA was reverse transcribed to cDNA using the PrimeScript RT reagent Kit (Takara, China, RR047A). Quantitative real‐time PCR was carried out using TB Green Premix Ex Taq (Takara, China, RR420A) to amplify the cDNA templates. Primer sequences are listed in Table S2.

### 5.11 Dual Luciferase Reporter Assay

The wild‐type and mutant 3’‐UTR fragments of G3BP1 were inserted downstream of the firefly luciferase gene in the psiCHECK‐2 vector. Cells were seeded in 24‐well plates and co‐transfected with 500 ng of each reporter plasmid and 50 nM miR‐197‐3p mimic or negative control using Lipofectamine 3000. After 48 h, firefly and Renilla activities were measured with the Dual‐Luciferase Reporter Assay System (Promega, USA) on a microplate reader (Synergy Neo2, BioTek, USA). Relative luciferase activity was calculated as the ratio of firefly to Renilla signals and normalised to the negative control group.

### 5.12 Isolated Culture of Primary Mouse Articular Chondrocytes

Articular cartilage was extracted from the femoral condyle and tibial plateau of 2‐day‐old C57BL/6 mice. The cartilage was cut into 1 mm^3^ pieces and digested with 0.25% trypsin for 30 min, followed by digestion with 0.2% type II collagenase at 37°C for 9 h. The released chondrocytes were collected by filtration through a 100 µm filter and washed with PBS. The cells were then cultured in DMEM supplemented with 10% fetal bovine serum and 1% penicillin‐streptomycin.

### 5.13 Cell Treatment

Primary chondrocytes were stimulated with IL‐1β (10 ng/mL) for 48 h to establish an in vitro inflammatory model. For oxidative stress experiments, cells were treated with H_2_O_2_ (200 µM) for 24 h. For senescence induction, Doxo (100 nM) was applied for 9 days, with medium refreshed every 2 days.

### 5.14 Cell Live/Dead Assay

The cells were stained using the Live/Dead Cell Double Staining Kit (Abbkine, China, KTA1001). Briefly, chondrocytes were washed twice with PBS, then incubated with 0.2 mL of staining solution containing 0.2 µL LiveDye and 0.2 µL NucleiDye at 37°C for 15 min. After incubation, the cells were washed twice with PBS. The samples were then observed under a laser confocal microscope, with live cells showing green fluorescence and dead cells showing red fluorescence.

### 5.15 Cell Proliferation Assay

Chondrocyte viability was assessed using the Cell Proliferation Kit (CCK‐8; Abbkine, China, BMU106‐CN). Chondrocytes were seeded in a 96‐well plate and treated with CCK‐8 working solution in the dark for 2 h on days 1, 3, and 5. Absorbance was then measured at 450 nm using a microplate reader (SynergyNeo2, BioTek, USA).

### 5.16 ROS Detection

ROS generation was assessed using Reactive Oxygen Species Assay Kit (Beyotime, China, S0033S). After incubating the chondrocytes for 48 h with or without H_2_O_2_ at 37°C, the cells were stained with 10 µM DCFH‐DA for 20 min. Following staining, the cells were washed with PBS and observed under a fluorescence microscope to visualize ROS production. For flow cytometry analysis, cells were harvested after staining and mean fluorescence intensity was measured to quantify ROS levels.

### 5.17 SA‐β‐Gal Staining

The chondrocytes were seeded in 12‐well culture plates and cultured until they reached 70–80% confluence. The cells were then stained for senescence using the β‐galactosidase Staining Kit (Solarbio, China, G1580). After washing the cells twice with PBS, they were fixed, followed by the addition of β‐Gal staining solution and incubation at 37°C overnight. After washing with PBS, the cells were observed under a light microscope.

### 5.18 Apoptosis Detection

Apoptosis of chondrocytes was assessed using the Annexin V‐YSFluor 647/PI Apoptosis Detection Kit (Yisheng, China, 40304ES50). After digestion and washing with PBS, the cells were resuspended in 100 µL 1× Binding Buffer, then stained with 5 µL Annexin V‐YSFluor 647 and 10 µL PI. The cells were incubated at room temperature for 15 min, protected from light. After incubation, 400 µL 1× Binding Buffer was added to the cells, and they were analyzed by flow cytometry.

### 5.19 Metabolic RNA‐seq Analysis

Cells from the control and miR/PBNP@Gel groups were harvested and total RNA was isolated with TRIzol Reagent (Invitrogen, USA). RNA integrity was verified on an Agilent 2100 Bioanalyzer with RIN values above 7.0. Poly(A) enriched libraries were prepared using the NEBNext Ultra II RNA Library Prep Kit and sequenced on an Illumina NovaSeq 6000 platform to generate 150 bp paired end reads. Adaptor trimmed reads were quality filtered with Trimmomatic and aligned to the Rattus norvegicus reference genome Rnor 6.0 using HISAT2. Gene counts were obtained with featureCounts and differential expression between the two groups was assessed in R version 4.2.0 with DESeq2. Transcripts with adjusted *p* values below 0.05 and absolute log_2_ fold change values of at least 2 were considered significant. Metabolic pathway enrichment of the differentially expressed genes was evaluated with the KEGG database using the clusterProfiler package.

### 5.20 Mitochondrial Membrane Potential Assay

Mitochondrial membrane potential was measured using the JC‐1 Mitochondrial Membrane Potential Assay Kit (Yisheng, China, 40706ES60). Cells were incubated with 1 mL JC‐1 working solution at 37°C for 20 min, washed twice with 1 × buffer and imaged by fluorescence microscopy. Red fluorescence (JC‐1 aggregates) indicates intact membrane potential, while green fluorescence (JC‐1 monomers) indicates membrane depolarisation.

### 5.21 Mitochondrial Morphology Imaging

Mitochondria were stained with PK Mito Deep Red (Warbio, China, PKMDR‐1) for structured illumination microscopy and with MitoTracker Green FM (Yisheng, China, 40742ES50) for confocal laser scanning microscopy.

### 5.22 Mitochondrial Respiration

Oxygen consumption rate experiments were performed using a Seahorse XF Cell Mito Stress Test Kit (Agilent Technologies, USA, 103015–100) in a Seahorse XF96 Flux Analyzer (Agilent Technologies, USA, 102416‐100). Cells were seeded at least six replicates for each condition in DMEM to reach 90% confluence at the day of measurement. Medium was completely replaced with reconstituted Seahorse DMEM (Agilent Technologies, USA) with 10 mM glucose, 2 mM glutamine and 1 mM pyruvate adjusted to pH 7.4 and incubated for 60 min at 37°C in a CO_2_‐free incubator before measurements.

### 5.23 Isocitrate Dehydrogenase (IDH) Activity Assay

The activity of IDH was measured using a colorimetric IDH Assay Kit (Abcam, UK, ab102528) according to the manufacturer's instructions. Briefly, chondrocytes were harvested after the indicated treatments and lysed in assay buffer on ice. The lysates were centrifuged at 12 000 g for 10 min at 4°C, and the supernatant was collected for analysis. The reaction was initiated by adding the reaction mix, and the increase in absorbance was measured at 450 nm using a microplate reader. IDH activity was calculated based on the rate of colorimetric change and normalized to total protein content.

### 5.24 ATP Quantification

Cellular ATP was measured with the Enhanced ATP Assay Kit (Beyotime, China, S0027). Cell lysates were mixed with the ATP detection working solution, and chemiluminescence was read on a microplate reader (SynergyNeo2, BioTek, USA).

### 5.25 Rat Model of Osteoarthritis

3‐month‐old male Sprague‐Dawley rats were anesthetized with an intraperitoneal injection of pentobarbital sodium (50 mg kg^−^^1^). A medial parapatellar incision was made in the right knee, the patella was gently dislocated laterally and the joint was flexed to expose the anterior cruciate ligament. The ligament was completely transected at its tibial attachment with microsurgical scissors, and successful transection was confirmed by a positive anterior drawer test. The joint space was rinsed with sterile saline, the patella was relocated and the joint capsule and skin were closed in layers with absorbable sutures. Sham‐operated rats underwent the same exposure without ligament transection.

After successful model establishment, animals except those in the sham group were randomly assigned to different treatment groups. Histological and micro‐CT analyses were performed by investigators blinded to the group allocation. For intra‐articular treatment, chemically modified miR‐197‐3p agomir was incorporated into hydrogel or PBNP and injected into the knee joint at a dose of 5 µg miR‐197‐3p in a total volume of 10 µL. Animals were allowed free cage activity under standard housing conditions and were harvested at predefined time points for evaluation of osteoarthritic changes.

### 5.26 Gait Analysis

In this experiment, gait analysis of rats was performed using the DigiGait treadmill (Mouse Specifics, Inc., USA). Prior to testing, each rat underwent a 5‐day adaptation period on the treadmill to help it acclimate to the device. During training, each rat was allowed a maximum of 1 min of treadmill exercise per session, with the speed set between 10 and 25 cm/s. Each training session required the rat to complete at least three continuous stride cycles to ensure acclimation to the treadmill and achieve a stable gait.

### 5.27 Micro Computed Tomography (micro‐CT)

Rat knee joints were scanned with a high‐resolution micro‐CT system (µCT45, SCANCO Medical, Switzerland). Coronal and sagittal datasets were acquired at 70 kVp, 114 µA and 8 W, followed by 3D reconstruction using the manufacturer's software. Standard trabecular morphometric parameters were then quantified.

### 5.28 Histologic Examinations

Rat knee joints were fixed in 4% paraformaldehyde for 24 h, decalcified in 10% EDTA (pH 7.4) for 2 weeks, dehydrated through graded ethanol and embedded in paraffin. Coronal sections 5 µm thick were cut with a rotary microtome, mounted on glass slides, baked, deparaffinised and rehydrated. Sections were stained with haematoxylin and eosin to assess general morphology and with safranin O/fast green solution (0.1% safranin O, 0.01% fast green; Solarbio, China) to evaluate proteoglycan loss. Stained slides were imaged using a digital slide scanner (3D histech, 3DHISTECH, Hungary) for subsequent analysis.

### 5.29 Immunohistochemical Analysis

Paraffin sections were deparaffinised and rehydrated, then microwaved in citrate buffer (pH 6.0) for 10 min to retrieve antigens. Endogenous peroxidase was blocked with 3% H_2_O_2_ for 10 min at room temperature, followed by 5% bovine serum blocking for 30 min. Sections were incubated at 4°C overnight with primary antibodies against Col2a1 (1:100, Abcam, UK, ab307674), Mmp13 (1:100, Proteintech, USA, 18165‐1‐AP), Aggrecan (1:100, Abcam, UK, ab313636), and P21 (1:50, Cell Signaling Technology, USA, #37543). After washing, an HRP‐conjugated secondary antibody was applied for 1 h at room temperature, and color was developed with DAB. Slides were counterstained with haematoxylin, dehydrated, cleared, and mounted. Images were captured using a digital slide scanner (3D histech, 3DHISTECH, Hungary).

### 5.30 Western Blot Analysis

Proteins were extracted from rat knee tissue using RIPA lysis buffer (Applygen, China, C1053) on ice, and concentrations were quantified with an Enhanced BCA Protein Assay Kit (Beyotime, China, P0010). Equal protein amounts were resolved on 15% SDS‐PAGE gels and transferred to 0.45 µm PVDF membranes (Merck Millipore, USA, ISEQ00010). Membranes were blocked with 5% non‐fat milk for 1 h at room temperature, then incubated overnight at 4°C with primary antibodies against P16 (1:1000, Cell Signaling Technology, USA, #29271) and p21 (1:1000, Cell Signaling Technology, USA, #37543). After TBST washes, HRP‐conjugated secondary antibody (1:5000, Cell Signaling Technology, USA) was applied for 1 h at room temperature. Bands were visualised with ECL ultra‐sensitive luminescent liquid (LABLEAD, China, E1060) and imaged on a Chemical imaging System (Chemidoc Touch, Bio‐Rad, USA).

### 5.31 Statistical Analysis

All in vitro experiments were performed using independent biological replicates. Unless otherwise specified, three biological replicates per group were included (*n* = 3). For Seahorse metabolic flux analyses, six independent biological replicates per group (*n* = 6) were used to ensure robust assessment of mitochondrial function. Animal experiments were conducted with four animals per group (*n* = 4), as determined based on prior studies and preliminary data. For quantitative assays, each biological replicate was measured in technical triplicates. Data are presented as mean ± SEM. Differences between two groups were analyzed with a two‐tailed unpaired Student's t‐test. For comparisons involving three or more groups, one‐way analysis of variance was applied followed by Tukey's post hoc test. Statistical analyses and graphing were performed using GraphPad Prism 8.0, and *p* < 0.05 was regarded as statistically significant.

## Author Contributions

X.C. and Z.L. jointly conceived, designed and executed the experimental work of this study, including in vitro and in vivo experiments, data analysis and wrote the manuscript draft. C.Z. was responsible for material synthesis. X.Y. assisted with the cell and animal experiments. R.W. and Y.B. assisted with data analysis, while J.X. and H.W. contributed to manuscript revisions. Y.W. and L.B. contributed to manuscript revisions. J.S. and X.W. were responsible for project supervision and funding acquisition. All authors provided valuable feedback and contributed to the manuscript revisions.

## Funding

This work was funded by Beijing Municipal Natural Science Foundation (Grant ID: 7232129), National Natural Science Foundation of China (Grant ID: 82572715), Special Project for Research and Development in Key areas of Guangdong Province (2023B1111050003), the 2025 Undergraduate Innovation Training Program of Peking Union Medical College, university‐level project (Grant ID: 2025dcxm132), National High Level Hospital Clinical Research Funding (Grant ID: 2025‐PUMCH‐A‐055), and Non‐profit Central Research Institute Fund of Chinese Academy of Medical Sciences (Grant ID: 2025‐JKCS‐04).

## Patient Consent for Publication

Not applicable. This study does not contain individual patient case details, patient images, or any patient‐identifiable information.

## Conflicts of Interest

The authors declare no conflicts of interest.

## Supporting information




**Supporting File**: advs76364‐sup‐0001‐SuppMat.docx.

## Data Availability

The data that support the findings of this study are available on request from the corresponding author. The data are not publicly available due to privacy or ethical restrictions.
