# Supplementary material for: Nanozyme‐Reinforced miR‐197‐3p Delivery Resets Metabolic and Senescence Pathways to Rejuvenate Osteoarthritic Cartilage
Source: Adv Sci (Weinh). 2026 Jul 4;13(53):e76364. doi: 10.1002/advs.76364 (PMC13334597; doi:10.1002/advs.76364)
Supplement: Supplementary file 1 [file ADVS-13-e76364-s001.docx]

**Supplementary Information**

**Nanozyme-reinforced miR-197-3p delivery resets metabolic and senescence pathways to rejuvenate osteoarthritic cartilage**

**Xuejie Cai, Zehui Lv, Xingdong Yang, Chen Zhang, Ruoying Wang, Yixin Bian,** **Jiawei Xu, Han Wang, Yingjie Wang, Long Bai, Jiacan Su and Xisheng Weng**

**Fig. S1.** G3BP1 overexpression attenuates the protective effects of miR-197-3p in IL-1β-treated chondrocytes (n = 3). Data are presented as mean ± SEM. One-way ANOVA with Tukey’s post hoc test was used for multiple comparisons. *** P<0.001, **** P<0.0001.

**Fig. S2.** G3BP1 knockdown partially mimics the effects of miR-197-3p in IL-1β-treated chondrocytes (n = 3). Data are presented as mean ± SEM. One-way ANOVA with Tukey’s post hoc test was used for multiple comparisons. * P<0.05, ** P<0.01, *** P<0.001, **** P<0.0001.

**Fig. S3.** Representative immunofluorescence images showing G3BP1 puncta formation in chondrocytes under different treatments (scale bar: 10 µm).

**Fig. S4.** Size of PBNPs in GelMA.

**Fig. S5.** Zeta potential of PBNPs in GelMA.

**Fig. S6.** X-ray diffraction of PBNPs in GelMA.

**Fig. S7.** Quantitative characterization of miRNA encapsulation efficiency (EE) and loading content (LC) in miR@Gel and miR/PBNP@Gel (n = 3). Data are presented as mean ± SEM. One-way ANOVA with Tukey’s post hoc test was used for multiple comparisons. * P<0.05, ** P<0.01.

**Fig. S8.** In vitro cumulative release of miR-197-3p from miR@Gel and miR/PBNP@Gel in simulated synovial fluid over 14 days (n = 3).

**Fig. S9.** RNase protection assay of miR-197-3p (n = 3).

**Fig. S10.** Gating strategy for ROS detection in flow cytometry.

**Fig. S11.** Representative fluorescence images of ROS staining in chondrocytes following different treatments (scale bar: 200 µm).

**Fig. S12.** Representative images of SA-β-Gal staining in chondrocytes following different treatments (scale bar: 50 µm).

**Fig. S13.** Gating strategy for apoptosis analysis by Annexin V/PI staining.

**Fig. S14.** Representative JC-1 staining images showing mitochondrial membrane potential under different treatments (scale bar: 10 µm).

**Fig. S15.** Schematic illustration of Seahorse XF analysis of mitochondrial function.

**Fig. S16.** IDH activity in chondrocytes under oxidative stress and treatment conditions (n = 3). Data are presented as mean ± SEM. One-way ANOVA with Tukey’s post hoc test was used for multiple comparisons. * P<0.05, ** P<0.01.

**Fig. S17.** Semi-quantitative analysis of cristae number per mitochondrion(n = 3). Data are presented as mean ± SEM. One-way ANOVA with Tukey’s post hoc test was used for multiple comparisons. * P<0.05, ** P<0.01.

**Fig. S18.** In vivo biosafety evaluation of engineered exosome treatment.

(A) Blood routine and serum biochemical parameters showing no significant systemic toxicity after treatment (n = 3). Data are presented as mean ± SEM. One-way ANOVA with Tukey’s post hoc test was used for multiple comparisons. ns, not significant.

(B) Histological analysis of systemic toxicity in rats. The major organs (heart, liver, spleen, lung and kidney) of SD rats in the indicated groups were collected after a 8-week treatment for H&E staining (scale bar: 20 μm).

**Fig. S19.** Representative IVIS images of Cy5-labeled miR-197-3p, miR@Gel, and miR/PBNP@Gel at 1, 7, and 14 days post intra-articular injection.

**Fig. S20.** Therapeutic effects in an age-related osteoarthritis model in guinea pigs.

(A) Representative micro-CT images showing joint structural changes following different treatments.

(B) Representative H&E and SO&FG staining of articular cartilage.

**Table S1.** Top 10 differentially expressed metabolites in OA cartilage after miR/PBNP@Gel treatment. Metabolites are ranked by fold change, calculated from the mean abundance of three biological replicates per group. Data are presented as mean ± SEM. Two-tailed Student’s t-test was used for comparison.

**Table S2.** Primer sequences used for qRT-PCR in this study.
